# LAP2α drives breast tumorigenesis by mitigating replication stress

**DOI:** 10.1038/s41419-026-08433-6

**Published:** 2026-02-03

**Authors:** Yanhui Ma, Yan Qin, Peida Bao, Ao Wei, Zhenzhen Yang, Ling Liu, Shuai Liu, Roland Foisner, Lei Shi, Qi Zhang, Kaiwen Bao

**Affiliations:** 1 Key Laboratory of Breast Cancer Prevention and Therapy (Ministry of Education), Key Laboratory of Immune Microenvironment and Disease (Ministry of Education), The Province and Ministry Co-sponsored Collaborative Innovation Center for Medical Epigenetics, School of Basic Medical Sciences, Tianjin Medical University Cancer Institute and Hospital, Tianjin Medical University, Tianjin, China; 2 Department of Clinical Laboratory, The People’s Hospital of Yongcheng, Shangqiu, Henan China; 3 Max Perutz Labs, Vienna Biocenter Campus (VBC), Vienna, Austria; 4 Medical University of Vienna, Max Perutz Labs, Vienna, Austria; 5 Department of Clinical Laboratory, First Affiliated Hospital of Xi’an Jiaotong University, Xi’an, Shaanxi China

**Keywords:** Breast cancer, DNA, Breast cancer

## Abstract

Replication protein A (RPA) plays a vital role in replication stress response, with RPA-coated single-stranded DNA (ssDNA) acting as a critical platform for the coordination of the genome surveillance machinery. In previous studies, we reported that the lamin-associated protein LAP2α interacts physically with RPA, aiding its localization to damaged chromatin for genome protection. However, the significance of the LAP2α-mediated RPA deposition in tumor progression remains unclear. Here, we reveal that LAP2α promotes breast tumorigenesis by counteracting replication stress-induced DNA damage. Furthermore, we demonstrate that defects in RPA loading caused by LAP2α deficiency slow breast tumor growth and sensitize tumors to chemotherapeutic treatments. In addition, we found that LAP2α could directly stimulate the loading of RPA onto ssDNA. Collectively, our study characterizes a critical role of LAP2α-enhanced RPA loading in promoting breast tumorigenesis and positions the LAP2α-RPA complex as a promising target for therapeutic intervention in breast cancer.

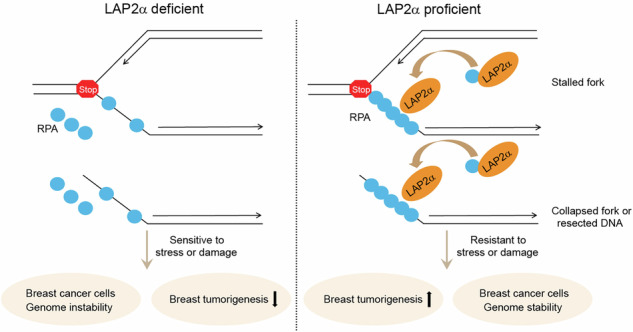

## Introduction

Breast cancer remains one of the most prevalent and lethal malignancies globally, representing a substantial public health challenge due to its high incidence, mortality rates, and its negative effects on patients’ quality of life [1, 2]. The onset and progression of breast cancer are driven by intricate molecular mechanisms that disrupt genome stability and cellular homeostasis, leading to uncontrolled cell proliferation, evasion of apoptosis, and metastatic dissemination [3]. A comprehensive understanding of the molecular mechanisms underlying breast cancer, particularly the interplay between genome instability and cellular stress responses, is critical for developing novel therapeutic strategies and enhancing patient outcomes [4, 5].

In contrast to normal cells, cancer cells with active oncogenes or faulty tumor suppressor genes often show irregular cell cycle progression, unregulated DNA replication, and increased replication stress [6–8]. This characteristic renders cancer cells particularly dependent on the replication stress response pathways, which also provides a promising target for therapies that selectively destroy these cells while sparing normal cells [9]. When faced with replication stress, replication forks can stall, resulting in the creation of extensive regions of single-stranded DNA (ssDNA) that become coated with replication protein A (RPA) [10, 11]. Prolonged stalling or failure to restart will potentially lead to fork collapse and DNA double-strand break (DSB) generation [12, 13].

The serine/threonine kinase Ataxia telangiectasia and Rad3-related (ATR) is a key regulator of the DNA damage response (DDR) that gets activated by ssDNA-RPA, which is an early responder to replication stress [1, 14]. Once activated, ATR works with downstream effectors to slow down cell cycle progression and stabilize stalled replication forks, reducing the impact of replication stress and aiding DNA repair [14]. Inactivating ATR has been identified as a promising cancer treatment strategy, and studies indicate that inhibiting RPA alone can also heighten replication stress and suppress tumor growth [15, 16]. These insights underscore the critical role of the ssDNA-RPA-dependent surveillance network in maintaining genome stability and highlight its potential as a target for cancer therapy [17].

LAP2α, the largest isoform produced by the LAP2 gene (TMPO), interacts with A-type lamins and plays a key role in the localization of lamin A/C within the nucleoplasm [18]. This interaction influences retinoblastoma protein (pRb)-mediated gene expression regulation, as well as progenitor cell proliferation and differentiation in highly regenerative tissues [19]. Notably, LAP2α is highly expressed in various human tumors, consistent with its elevated expression in proliferating cells [20]. In cases of follicular lymphoma and multiple myeloma, elevated levels of LAP2α have been linked to worse clinical outcomes, indicating its potential involvement in tumor development and its promise as a prognostic marker [21, 22]. Currently, it is believed that LAP2α’s role in the regulation of the cell cycle via lamin A/C and pRb is a significant factor in its potential contribution to tumorigenesis [23].

In our previous study, we reported that LAP2α physically interacts with replication protein A (RPA), enhancing RPA’s deposition on damaged chromatin through these interactions [24]. Importantly, the binding of RPA to ssDNA promoted by LAP2α is crucial for the protection of replication forks, activation of ATR, and repair of damaged DNA [24]. Nevertheless, it remains insufficiently investigated whether LAP2α-promoted genome surveillance contributes to tumor progression.

Here, we report that elevated LAP2α expression in breast cancer correlates with adverse clinical prognosis. Genetic ablation of LAP2α significantly attenuates breast tumor progression, a phenotype mechanistically linked to impaired RPA deposition and consequent DNA damage accrual in neoplastic cells. Notably, depleting of LAP2α or disrupting the LAP2α-RPA molecular interface synergistically enhances chemotherapeutic responses. Furthermore, through biochemical reconstitution assays, we demonstrate that LAP2α directly stimulates RPA loading onto ssDNA, thereby elucidating the mechanistic basis for its role in promoting replication stress resilience.

## Materials and methods

### Cell culture

U2OS and MDA-MB-231 cells were purchased from ATCC (Manassas, VA) and cultured under the manufacturer’s instructions. All of the cells were cultured in DMEM supplemented with 10% fetal bovine serum and 1% penicillin–streptomycin and maintained in 5% CO_2_ at 37 °C. All of the cells were authenticated by examination of morphology and growth characteristics and confirmed to be mycoplasma-free.

### Antibodies and reagents

The sources of antibodies against the following proteins or post-translational modifications were as follows: γH2AX (9718S, for IHC), CHK1 pS345 (2341S, for WB) and CHK1 (2360S, for WB), from Cell Signaling Technology; RPA2 pS33 (A300-246A, for WB) from Bethly Lab; FLAG (F3165, for WB) from Sigma; γH2AX (05-636, for WB) from Millipore; LAP2α (ab5162, for WB and human tissue IHC), Ki-67 (ab16667, for mouse tissue IHC), CldU/BrdU (ab6326, for IF), RPA2 (ab2175, for WB and IF), and RPA1 (ab176467, for WB) from Abcam; β-actin (AC004, for WB) from Abclonal; RPA2 (10412-1-AP, for WB) from Proteintech; 53BP1 (NB100-304, for IF) from NOVUS; IdU/BrdU (347580, for IF) from BD Bioscience; H2AX (YT2155, for WB) from Immunoway; and LAP2α (IQ175, for mouse tissue IHC) from ImmuQuest. Hydroxyurea (H8627), IdU (I7125), CldU (C6891), DAPI (F6057), and 4-OHT (H6278) were purchased from Sigma. Rucaparib (AZD2281) and cisplatin (S1166) were purchased from Selleck. Aphidicolin (HY-N6733) was purchased from MedChemExpress.

### Immunofluorescence

Cells cultured on glass coverslips (BD Biosciences) were first treated with 0.5% Triton X-100 for 5 min on ice to isolate non-chromatin fractions. Subsequently, they were fixed in a solution of 3% paraformaldehyde and 2% sucrose for 15 min at room temperature. Following fixation, the cells underwent permeabilization using 0.5% Triton X-100 for an additional 5 min on ice, after which they were incubated in a blocking buffer composed of 0.1% Triton X-100 and 5% donkey serum in PBS for 1 h at room temperature. The next step involved staining the cells with the corresponding primary and secondary antibodies conjugated to either Alexa Fluor 488 or 647 (Invitrogen). Confocal imaging was performed using a Zeiss LSM 900 microscope equipped with a ×63 oil objective. To avoid bleed-through in double-staining experiments, each fluorescent dye was scanned separately in a multi-tracking mode.

### RNA interference

All siRNA transfections were performed using Lipofectamine RNAi MAX (Invitrogen) following the manufacturer’s recommendations. The final concentration of the siRNA molecules is 10 nM and cells were harvested 96 h later according to the purposes of the experiments. Control siRNA (ON-TARGETplus Non-Targeting Pool, D-001810-10) was obtained from Dharmacon in a smart pool manner, while siRNAs of LAP2α(siRNA-1: GUCUAGAAGUGGCUAAGCA, siRNA-2 targeting 3’ UTR: GCUUUCUAGAUCACAUAUU) were chemically synthesized by Sigma (Shanghai, China). Targeting sequence against LAP2α 3’UTR (GCTTTCTAGATCACATATTAG) was cloned into pLKO.1 vector to generate LAP2α stably knockdown cells.

#### EMSA

DNA substrates labeled at the 5’ end with Cy3 (Invitrogen) at a concentration of 5 nM were mixed with specified amounts of proteins and incubated at room temperature for 20 min in a 1× binding buffer composed of 25 mM Tris (pH 7.5), 200 mM NaCl, 5 mM MgCl_2_, 1 mM DTT, 5% glycerol, and 0.05% Triton X-100. Following this, the total reaction volume of 20 L was combined with 2 L of 10× loading dye and subjected to electrophoresis in a 4% native acrylamide/Bis gel using cold 0.5× TBE buffer (which contains 44.5 mM Tris, 44.5 mM boric acid, and 0.5 mM EDTA, pH 8.3). Signal detection was performed with an Alliance Q9 imager, and band intensities were analyzed using ImageJ (NIH). Additionally, gel shift assays utilizing 5’ biotin-labeled ssDNA (GCTTGCATGCCTGCAGGCCAGCCTCAATCTCATC, at 10 nM) were conducted under the same conditions. After electrophoresis, the gels were transferred onto a nylon membrane, and DNA-protein interactions were assessed using a LightShift™ Chemiluminescent EMSA Kit (Thermo Scientific). Detection of signals was carried out with a Tanon-5200 imager (Tanon). LAP2α and RPA were purified and reconstituted as previously described [24, 25].

#### BRET

Bioluminescence resonance energy transfer (BRET) assay was used to monitor DNA conformations upon RPA binding. A microplate reader (TECAN SPARK) was used to excite Cy3 and observe Cy3 and Cy5 emission. The Cy3 excitation wavelength was set to 530 nm, and emission was measured at 565 nm. Cy5 excitation was caused by the energy transfer from Cy3, and its emission was measured at 660 nm. Excitation and emission slit widths were all set to 10 nm. Experiments were carried out in reaction buffer (50 mM Tris–HCl, pH 7.5, 5 mM MgCl_2_, 100 mM NaCl, 1 mM DTT, and 0.1 mg/ml BSA) with 10 nM dT30 ssDNA at 25 °C.

### ssDNA-Pull down

A total of 30 pmol of biotin-labeled 70-nucleotide single-stranded DNA (TGCAGCTGGCACGACAGGTTTTAATGAATCGGCCAACGCGCGGGGAGAGGCGGTTTGCGTATTGGGCGCT) was preincubated with 30 L of streptavidin Sepharose beads in PBS while rotating at 4 °C overnight. Following this, one-third of the biotin-labeled ssDNA was mixed with nuclear extracts in NETN buffer (0.2% NP40, 150 mM NaCl, 2 mM EDTA and 50 mM Tris-HCl, pH 7.5) and incubated at 4 °C for 8 h or at room temperature for 1 h, after which washing and immunoblotting were performed.

### Pull-down assay with oligonucleotide titrations

His-LAP2α protein was incubated with Ni-NTA beads. The resulting LAP2α protein-Ni-NTA beads complexes were then incubated with RPA protein. After five washes, one-eighth of the beads were aliquoted and incubated with 1-, 5-, and 20-fold molar excesses of dT10 or dT30 ssDNA solution, respectively at room temperature for 1 h. After washing, the levels of RPA bound to LAP2α were assessed through immunoblotting.

### DNA fiber

To assess fork degradation, cells were initially labeled with IdU (25 M) for 20 min, followed by two washes with media, and then labeled with CldU (200 M) for another 20 min. After washing, the cells were treated with 4 mM HU ±100 M mirin for 4 h. Subsequently, the cells were trypsinized and resuspended in PBS to achieve a concentration of 7 ×10^5^ cells/ml. A mixture of 2 l of this cell suspension and 10 l of lysis buffer (200 mM Tris-HCl, pH 7.4, 50 mM EDTA, and 0.5% SDS) was placed on a clean glass slide. Following a 2-min incubation, the slides were tilted at a 15° angle, allowing the lysate to gradually flow down the slide. The slides were then air-dried, fixed using a 3:1 methanol/acetic acid solution, and treated with 2.5 M HCl for 80 min. Afterward, the slides were blocked with 5% BSA in PBS for 30 min and incubated overnight with anti-BrdU antibodies (BD Bioscience, 347580 for IdU, and Abcam, ab6326 for CldU) diluted in blocking buffer. After washing, secondary antibodies conjugated to Alexa Fluor 488 and 594 were diluted in PBS with 5% BSA and incubated with the cells at room temperature for 1 h. The slides underwent three washes with PBS. Following the washes, the cells were mounted using an anti-fade solution and examined under a Zeiss LSM 900 microscope with a 63× oil objective and processed with ZEN Blue software (Zeiss, v2.3). The lengths of all distinct fibers were measured using Image J software.

### Immunohistochemistry

Immunohistochemical analysis was conducted on paraffin-embedded human tissue microarrays (Ailina-Bio) or mouse tissues. In brief, tissue sections were heated on a panel at 65 °C for one hour, then deparaffinized using xylene and rehydrated through a series of ethanol dilutions. To inhibit endogenous peroxidase activity, sections were treated with 3% hydrogen peroxide in methanol for 30 min, after which they were washed three times for three minutes each with phosphate-buffered saline (PBS). The slides were then immersed in a 0.01 M citrate buffer solution (pH 6.0) and subjected to microwave heating for 30 min. Following another PBS wash, the sections were incubated in 10% normal goat serum for 30 min to block non-specific binding and then exposed to the primary antibody diluted in PBS with 10% normal goat serum overnight at 4 °C in a humidified chamber. Negative controls were established by substituting the primary antibody with preimmune rabbit serum (Sigma, S20-M). After washing three times for five minutes each with PBS, a peroxidase-conjugated secondary antibody (ZSGB-BIO) was applied for 30 min at room temperature, followed by three additional five-minute PBS washes. DAB solution (ZSGB-BIO) was then introduced, and the slides were counterstained with hematoxylin according to the manufacturer’s guidelines. Images were captured using an Olympus VS120 Slide Scanner. The tissue microarray (TMA) utilized in this research comprised 82 cases of tumor-adjacent normal breast tissue and 376 cases of breast carcinoma, which included 324 cases of invasive ductal carcinoma, 44 cases of invasive lobular carcinoma, and 8 cases of other histological types. Within the invasive ductal carcinoma cohort, there were 38 cases classified as Grade I, 195 as Grade II, and 91 as Grade III. Survival data were documented for 72 cases of luminal A, 43 cases of luminal B, 43 cases of Her2-enriched, and 35 cases of triple-negative breast carcinoma patients. The staining score for each specimen was determined by multiplying the extent score by the intensity score. Tumors collected from mice were fixed overnight in 10%-buffered formalin (Sigma, HT501128) and subsequently embedded in paraffin. Sections were stained with hematoxylin and eosin (H&E) or specific primary antibodies, followed by HRP-conjugated secondary antibodies and DAB visualization. The information of the human tissue microarray was provided in Supplementary Table S1.

### Primary tumor cell culture and re-inoculation procedure

PyMT mammary gland tumors were carefully isolated and processed under sterile conditions. Initially, any visible healthy tissue remnants were eliminated by thoroughly washing with an ample volume of PBS while shaking vigorously. The tumor tissue was then cut into smaller fragments using a scalpel in DMEM and further minced until they reached an approximate sizes of 1 mm³. Following this, the small tissue pieces were subjected to vigorous vortexing and washed with ten times their volume of PBS until no blood or debris was visible. The resulting tissue pellets were then re-suspended and incubated in a digestion buffer containing 160 g/ml collagenase and 25 g/ml hyaluronidase at 37 °C for one hour. Next, the mixture was filtered through cell strainers with gradually decreasing pore sizes down to 40 m. The dissociated specimens were sorted into single cells using EPCAM (BD Biosciences, #563478) via BD FACS Aria II cell sorter (BD Biosciences) and subsequently cultured in DMEM for additional experiments. For in vivo injection, the mammary gland area of female C57BL/6J mice was sterilized with 75% ethanol. Then 2 × 10^6^ cells were transplanted unilaterally into the fourth inguinal mammary fat pads of 8-week-old female C57BL/6J recipient mice. Tumor growth is monitored by measuring the length and wide with a digital caliper, and tumor volume is calculated as V = 1/6 × (π × length) × wide^2^.

### Cell survival assay

Cells were seeded into 96-well plates at a density of 2000 cells per well. After 24 h, the cells were exposed to different concentrations of genotoxic agents for a duration of 72 h. Subsequently, the Cell Titer Aqueous One Solution Reagent (G3582, Promega) was introduced to each well in accordance with the manufacturer’s guidelines. After incubating for one hour, cell viability was assessed by measuring the absorbance at 490 nm using a 550 Bio-Rad plate reader (Bio-Rad, Hertfordshire, UK).

### X-ray irradiation and laser micro-irradiation

IR was delivered by an X-ray generator (Radsource Corporation RS2000 PRO, 160 kV, 25 mA). Laser microdissection was performed with a UV-A laser (λ = 355 nm, 40% energy) using a Zeiss Observer.Z1 inverted microscope with a PALM MicroBeam laser microdissection workstation under 40× objective lens.

### Telomere FISH

Cells were treated with 0.1 g/ml colchicine (HY-16569, MedChemExpress) for 6 h to enrich cells at metaphase. Cells were trypsinized and resuspended in a hypotonic solution of 0.075 M KCl incubated at room temperature for 20 min. The cells were fixed twice with methanol: acetic acid (3:1) for 10 min each time as previously described. Alex488-labeled C-rich telomere probe (F1004, panagene) was used. Images were taken using a Zeiss LSM 900 microscope equipped with a ×63 oil objective.

### Flow cytometry analysis

To analyze the apoptosis rate, the cells were digested, stained with propidium iodide and Annexin V-FITC (CA1020, Solarbio), and incubated at room temperature for 10 min prior to flow cytometric analysis. To analyze the cell-cycle distribution, cells were harvested and fixed in cold ethanol overnight, treated with RNase A, stained with propidium iodide. All flow cytometry analyses were performed using a BD Biosciences LSRFortessa.

### Alkaline comet assay

Cells were embedded in an equal volume of 1% low-melting-point agarose and immediately pipetted onto slides pre-coated with 1% normal-melting-point agarose. The slides were then incubated in cold lysis solution (4250-050-01, R&D Systems) for 2 h, followed by a 20 min equilibration in alkaline electrophoresis buffer (300 mM NaOH, 200 mM EDTA). Electrophoresis was performed at 4 °C for 30 min at a constant current of 300 mA. Subsequently, the DNA was stained with 5 µg/ml propidium iodide for 30 min. Comet images were acquired using a fluorescence microscope and analyzed with CASP software.

### Mouse works

*Lap2*α conditional knockout mice were developed by Dr. Roland Foisner’s lab at Medical University of Vienna and University of Vienna, Austria. Neo selection marker was removed in our hands. PyMT-expressing *Lap2*α^flox/flox^;UbCre-ERT2 female mice were generated by crossing *Lap2*α^flox/flox^ mice with MMTV-PyMT transgenic mice (Jackson Laboratory) and UbiquitinC-Cre-ERT2 mice in C57BL/6J background. PyMT mammary tumors were monitored twice a week with a caliper, and tumor volume was calculated using the formula V = 1/6 × ( × length) × wide^2^. For *Lap2*α deletion, 4-OHT dissolved in 100% corn oil was injected intraperitoneally with 1 mg each day for five consecutive days when tumors reached approximately 100 mm^3^. At the experimental endpoint, when the largest tumor reached 1500 mm^3^ in diameter, animals were sacrificed. For chemotherapeutic treatments, when tumors reached approximate 200 mm^3^, *Lap2*α was knocked out and mice were treated with rucaparib (10 mg/kg, 2% v/v DMSO, 10% v/v HPBCD, 2-hydroxylpropyl-β-cyclodextrin, in PBS) per day or cisplatin (1 mg/kg, 5% v/v DMF, 40% v/v PEG300, 2% v/v TWEEN80 in H_2_O) once a week and the corresponding placebo. Tumors from the anterior mammary gland of each mouse in the same position were monitored and collected.

### Tumor Xenografts

MDA-MB-231 cells were plated and infected in vitro with lentiviruses carrying control shRNA or LAP2α shRNAs. Then, these cells (5 × 10^6^) were re-suspended with 200 l PBS and orthotopically transplanted onto the mammary fat pad of 6-week-old immunocompromised severe combined immunodeficiency (SCID) female mice. Once the tumor volume had reached approximately 100 mm^3^, mice within each group were randomly assigned into two subgroups. Half of the mice in each subgroup were treated with rucaparib (20 mg/kg, 2% v/v DMSO, 10% v/v HPBCD, 2-hydroxylpropyl-β-cyclodextrin, in PBS) and a corresponding placebo every 2 days. Each experiment utilized six mice per group. No blinding was done to group allocation. Mice were sacrificed at the experimental endpoint when the largest tumor grew to 2000 mm^3^. For the rescue experiments, LAP2α-knockdown cells were infected with lentivirus carrying LAP2α Wt or LAP2α/2RE followed by neomycin selection.

### Statistics

Data are reported as mean ± SEM when normality was assumed, or median as appropriate. Shapiro–Wilk test was used to test normality. A threshold of 0.05 was assumed. Equality of group variances was tested by Brown–Forsythe test, and Welch’s correction was further used when group variances were unequal. For comparisons between two groups, we performed Welch’s *t* test. For normally distributed data, the comparison of multiple groups was assessed by one-way ANOVA (for single-condition designs) or two-way ANOVA (for multiple-condition designs). For data set that did not meet normality test, Kruskal–Wallis ANOVA test with Dunn multiple-comparisons test (for comparisons between >two groups). A *P* value of less than 0.05 was defined as statistically significant.

### Study approval

All procedures involving animals were approved by the Ethics Committee of Tianjin Medical University. All experiments were performed in accordance with the approved protocol and other relevant guidelines and regulations.

## Results

### LAP2α is implicated in breast tumorigenesis

**Fig. 1:**
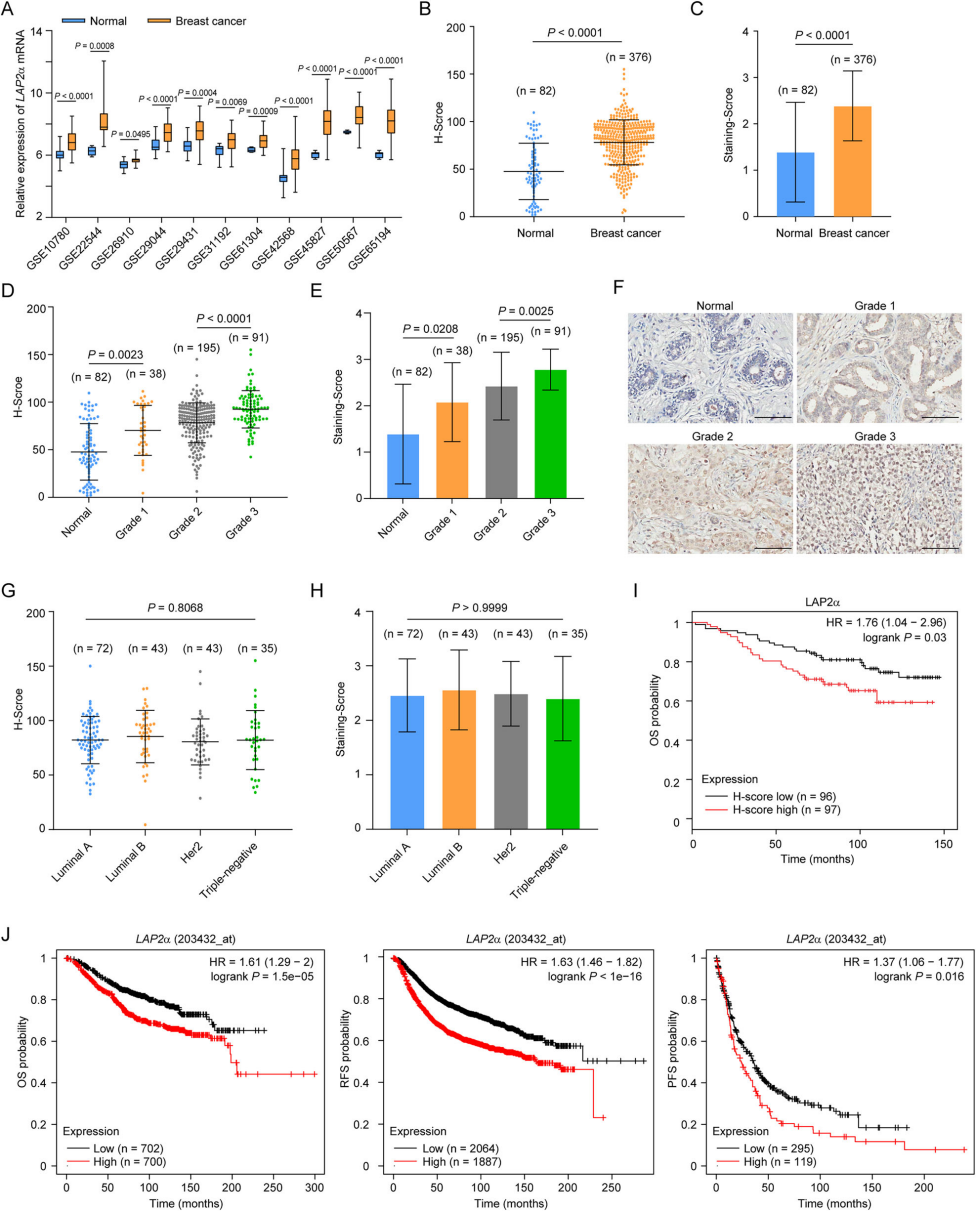
LAP2α is implicated in breast tumorigenesis. **A** Analysis of the expression of *LAP2*α in normal breast tissue and breast cancer samples from 11 independent data of Gene Expression Omnibus (GEO) with *LAP2*α (NM_003276) specific probe 203432_at. Data are shown as box plots. **B** Immunohistochemistry (IHC) analysis of LAP2α expression in tumor adjacent normal breast tissue and breast cancer samples. The expression level of LAP2α from IHC stainings was scored according to the staining intensity and extent. **C** Staining score defined by the H-Score from (**B**). **D** Quantitative analysis of the expression level of LAP2α in different grades of invasive ductal breast cancer samples according to IHC stainings. **E** Staining score corresponds to the H-Score presented in (**D**). **F** Representative images of different grades of invasive ductal breast cancer samples. **G** Quantitative analysis of the expression level of LAP2α across different molecular subtypes of invasive ductal breast cancer according to IHC stainings. **H** Staining score derived from the H-Score in (**G**). **I** Overall survival (OS) analysis of breast cancer patients according to the expression level of LAP2α from IHC stainings. **J** Survival analysis of breast cancer patients according to different *LAP2*α expression status with survival packages from KM plotter. OS overall survival, RFS relapse-free survival, PFS progression-free survival. Data are mean ± SDs for (**B**), (**C**), (**D**), (**E**), (**G**) and (**H**). Statistical tests were performed by Welch’s *t* test (**A**–**C**), one-way ANOVA with Dunnett’s multiple comparisons test (**D**, **E**, **H**) and one-way ANOVA with Tukey’s multiple comparisons test (**G**). Log-rank test for (**I**) and (**J**). Scale bar, 100 m.

Cancer cells often exhibit increased levels of replication stress, which are typically addressed through ssDNA-RPA-mediated DDR. In light of this, we aimed to investigate whether LAP2α-promoted RPA loading could contribute to tumor progression by responding to and alleviating endogenous replication stress. Analysis of data from 11 independent Gene Expression Omnibus (GEO) datasets with an LAP2α-specific probe revealed that the mRNA expression level of LAP2α is significantly elevated in breast cancer (Fig. 1A). Consistent with previous findings [26], overexpression of LAP2α was observed in liver, lung, colon, and thyroid cancers, with at least two GEO datasets supporting these findings (Supplementary Fig. S1A).

We subsequently conducted immunohistochemical staining (IHC) to analyze the expression of LAP2α in breast cancer samples and adjacent normal breast tissues. Quantitative analysis of the staining revealed that LAP2α expression is elevated in carcinoma samples (Fig. 1B, C and Supplementary Fig. S1B) and positively correlates with histological grade (Fig. 1D–F). Additionally, we observed that LAP2α is highly expressed, albeit to varying extents, in distinct histological subtypes of breast cancer (Supplementary Fig. S1C). Similar results were obtained when analyzing the Oncomine database that comprises gene expression and copy number data from 2000 breast tumors [4] (Supplementary Fig. S1E). Interestingly, the expression levels of LAP2α were similar across the luminal A, luminal B, HER2-enriched, and triple-negative breast cancer subtypes (Fig. 1G, H). Consistently, analysis of GEO datasets indicated that LAP2α expression showed only minor variation across different molecular subtypes of breast carcinoma (Supplementary Fig. S1F). These results suggest that dysregulation of LAP2α is commonly involved in breast carcinogenesis, regardless of the molecular subtype.

Next, we categorized breast cancer tissues into high and low LAP2α expression groups based on immunostaining scores. Notably, high LAP2α expression was associated with poor survival in breast cancer patients (Fig. 1I). Kaplan–Meier survival analysis of GEO datasets further corroborated that high LAP2α expression in breast carcinoma predicts unfavorable outcomes (Fig. 1J). Interestingly, the expression of RPA1 or RPA2 did not show significant changes in breast cancer when analyzed using The Cancer Genome Atlas (TCGA) and GEO datasets (Supplementary Fig. S1G, S1H), and Kaplan–Meier survival analysis did not reveal any correlation between these genes and patient outcomes (Supplementary Fig. S1I). In contrast, the expression and survival profiles of RPA3 were similar to those of LAP2α (Supplementary Fig. S1G–S1I). These findings suggest that the gain-of-function of LAP2α alone, rather than alterations in the abundance of RPA (which is already present in excess under normal conditions—6- to 10-fold more than the amount of ssDNA [14, 27]), may be sufficient for breast cancer cells to manage intrinsic replication stress. Alternatively, RPA3 may operate independently of RPA during breast cancer progression. Taken together, these results suggest that LAP2α is involved in breast tumorigenesis.

### Lap2α knockout suppresses the growth of mammary gland tumors

**Fig. 2:**
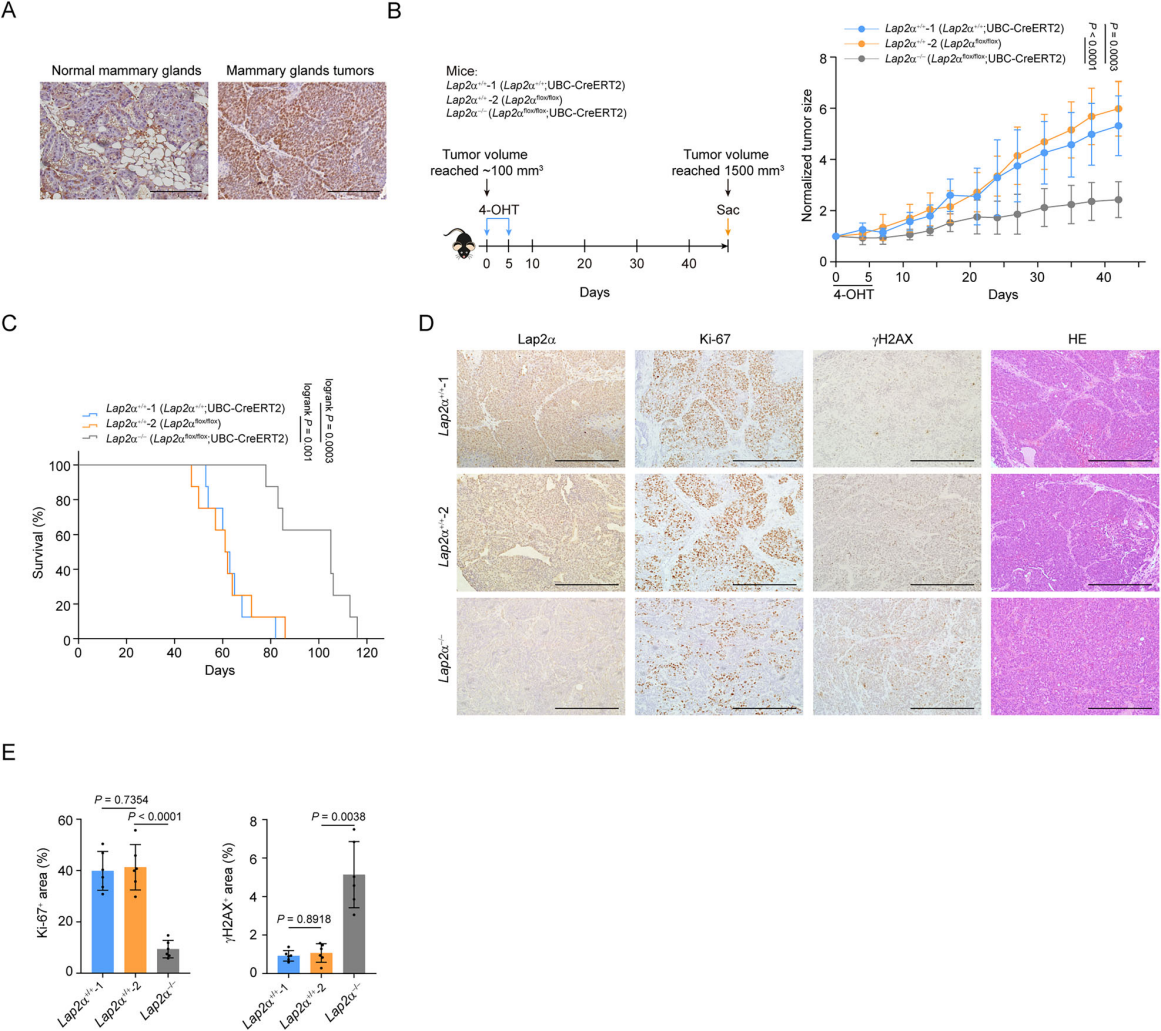
Lap2α knockout suppresses the growth of mammary gland tumors. **A** IHC analysis of Lap2α expression in tumor tissues from mice carrying the transgene PyMT and normal mammary gland tissue. **B** Mammary gland tumor growth of *Lap2*α^+/+^-1 (*Lap2*α^+/+^;UbCre-ERT2) or *Lap2*α^+/+^-2 (*Lap2*^flox/flox^) and *Lap2*α^−/−^ (*Lap2*α^flox/flox^;UbCre-ERT2) mice. Mice bearing PyMT tumors (*n* = 8) were injected with 4-OHT when tumor volume reached to 100 mm^3^ in size and the tumor growth was monitored for 40 days (one tumor in each mouse). Tumor size was normalized to the starting size of each tumor. **C** Kaplan–Meier overall survival analysis of *Lap2*α^+/+^ and *Lap2*α^−/−^ mice bearing PyMT tumors (*n* = 8). **D** IHC analysis of Lap2α, Ki-67 and γH2AX in mammary gland tumors from *Lap2*α^+/+^ and *Lap2*α^−/−^ mice. **E** Quantitative analysis of Ki-67^+^ area (*n* = 6) and γH2AX^+^ area (*n* = 6) in mammary gland tumors of *Lap2*α^+/+^ and *Lap2*α^−/−^ mice. Data are mean ± SDs for (**B**) and (**E**). Statistical tests were performed by two-way ANOVA with Tukey’s multiple comparisons test (**B**), log-rank test (**C**) and one-way ANOVA with Dunnett’s multiple comparisons test (**E**). Scale bar, 100 m.

To assess whether LAP2α plays a tumor-promoting role in breast cancer, we first examined Lap2α expression in mammary gland tumors from mice harboring the transgene encoding polyoma middle T (PyMT). Immunohistochemical (IHC) analysis revealed that LAP2α was highly expressed in tumor tissues compared to normal mammary glands (Fig. 2A). Next, we crossed PyMT mice with mice carrying the ubiquitin C-CreERT2 (UbCre) transgene and floxed alleles of the *Lap2*α*-*specific exon 4 in the *Lap2* gene (Supplementary Fig. S2A, S2B) [28, 29]. Once mammary tumors developed, the mice were treated with 4-hydroxytamoxifen (4-OHT) to activate Cre and induce *Lap2*α knockout (Supplementary Fig. S2B, S2C).

Tumor growth measurements revealed that *Lap2*α homozygous deletion led to a significant delay in tumor progression compared to PyMT-expressing *Lap2*α wild-type littermates, including *Lap2*α^+/+^UbCre or *Lap2*α^flox/flox^ (Fig. 2B). Survival analysis indicated that knockout of *Lap2*α resulted in improved survival for the mice with mammary tumors (Fig. 2C). Notably, the rapidly growing tumors in the later phase ultimately resulted in the survival of the *Lap2*α^−/−^ mice starts to decline at a similar rate after 80 weeks (Supplementary Fig. S2D). In agreement with the reduced tumor size, mammary tissue from *Lap2*α^−/−^ mice exhibited decreased cell proliferation and elevated DNA damage, as indicated by reduced Ki67 staining and increased γH2AX staining (Fig. 2D, E). These findings suggest that Lap2α loss leads to the accumulation of damaged DNA, thereby impairing tumor growth. Together, these results support the notion that Lap2α plays a critical role in promoting mammary cancer cell proliferation and survival.

### Lap2α knockout sensitizes mammary gland tumors to chemotherapeutic drugs

**Fig. 3:**
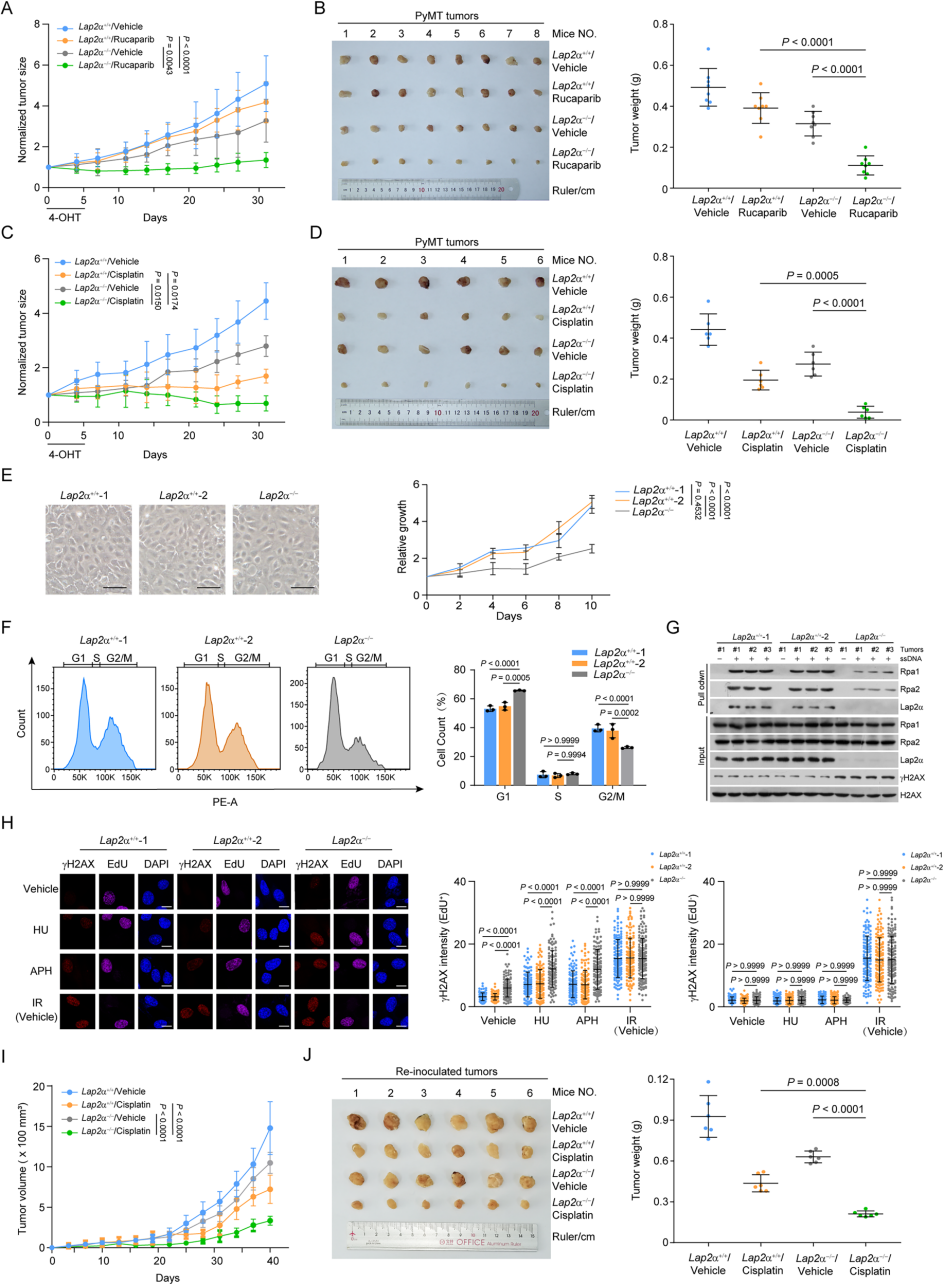
Lap2α knockout sensitizes mammary gland tumors to chemotherapeutic drugs. **A** Tumor growth of mock or rucaparib-treated *Lap2*α^+/+^ or *Lap2*α^−/−^ mice. Tumor size was normalized to the starting size of each tumor. **B** Tumor weight of mock or rucaparib-treated *Lap2*α^+/+^ or *Lap2*α^−/−^ mice at the endpoint (day 31). Each point represents an individual tumor from different mice (*n* = 8). **C** Tumor growth of mock or cisplatin-treated *Lap2*α^+/+^ or *Lap2*α^−/−^ mice. Tumor size was normalized to the starting size of each tumor. **D** Tumor weight of mock or cisplatin-treated *Lap2*α^+/+^ or *Lap2*α^−/−^ mice at the endpoint (day 31). Each point represents an individual tumor from different mice (*n* = 6). **E** Representative micrographs and quantitative analysis of mammary gland tumor cell growth of cultured mammary gland tumors from *Lap2*α^+/+^ and *Lap2*α^−/−^ mice (from biological triplicate experiments). **F** Representative flow cytometry data of the cell cycle and quantification of primary mammary tumor cells from *Lap2*α^+/+^ and *Lap2*α^−/−^ mice. **G** ssDNA pull-down analysis of RPA loading capacity in tumor cells derived from *Lap2*α^+/+^ and *Lap2*α^−/−^ mice. **H** Immunostaining and confocal microscopy analysis of γH2AX foci formation in tumor cells from *Lap2*α^+/+^ and *Lap2*α^−/−^ mice under HU (2 mM, 8 h), APH (0.5 M, 8 h), or 2 h post-IR (4 Gy) treatment (*n* > 100 from two independent experiments). **I** Re-inoculated tumor growth under mock or cisplatin treatment. Tumors from *Lap2*α^+/+^ or *Lap2*α^−/−^ mice were excised and digested to obtain single cells. After culturing for one generation, cells were injected onto the fat pad of C57BL/6J mice, and cisplatin was administered to tumor-bearing mice 8 days after tumor inoculation (*n* = 6). **J** Re-inoculated tumor weight under mock or cisplatin treatment. Tumors were collected from (**I**). Data are mean ± SDs for (**A**–**E**) and (**H**–**J**). Statistical tests were performed by two-way ANOVA with Tukey’s multiple comparisons test (**A**, **C**, **E**, **I**) and one-way ANOVA with Tukey’s multiple comparisons test (**B**, **D**, **F**, **J**, **H**). Scale bar: 100 m for (**E**) and 10 m for (**H**).

Since Lap2α contributes to replication stress response, DNA repair, and genome stability, we investigated whether targeting Lap2α would sensitize mammary tumors to additional DNA damage. Indeed, treatment of mice with either an ADP-ribose polymerase (PARP) inhibitor or cisplatin, two chemotherapeutic agents commonly used in cancer therapy, significantly suppressed tumor progression in *Lap2*α-knockout littermates (Fig. 3A–D). These results suggest that Lap2α-deficient mammary tumors are more vulnerable to additional DNA damage.

Given that UbCre is ubiquitously expressed in many tissues, we isolated and cultured PyMT-induced tumors from *Lap2*α^+/+^ and *Lap2*α^−/−^ mice to specifically investigate the role of Lap2α in tumor epithelial cells. These cells exhibited typical epithelial morphology, and *Lap2*α-knockout tumor cells grew at a slower rate (Fig. 3E). Accordingly, cell cycle analysis exhibited a G1/S arrest and a corresponding reduction in the G2/M-phase population in *Lap2*α-knockout tumor cells (Fig. 3F). Additionally, *Lap2*α ablation was associated with reduced RPA deposition and increased γH2AX levels (Fig. 3G). Knockout of *Lap2*α induced an enhanced H2AX signal in S-phase cells exposed to aphidicolin (APH) or hydroxyurea (HU) (Fig. 3H). In contrast, the γH2AX signal remained unaffected by *Lap2*α knockout in cells exposed to IR (Fig. 3H), further indicating that LAP2α deficiency may potentiate replication stress-associated DNA damage signaling. Importantly, we demonstrated that *Lap2*α-knockout tumors were more susceptible to cisplatin treatment when these tumor cells were re-inoculated into the mammary glands of C57BL/6 J mice (Fig. 3I, J). These findings suggest that the tumor growth retardation observed in *Lap2*α-knockout tumors may, at least in part, be attributed to defective RPA loading in mammary gland tumor cells.

### Lap2α acts intrinsically in promoting mammary gland tumor progression

**Fig. 4:**
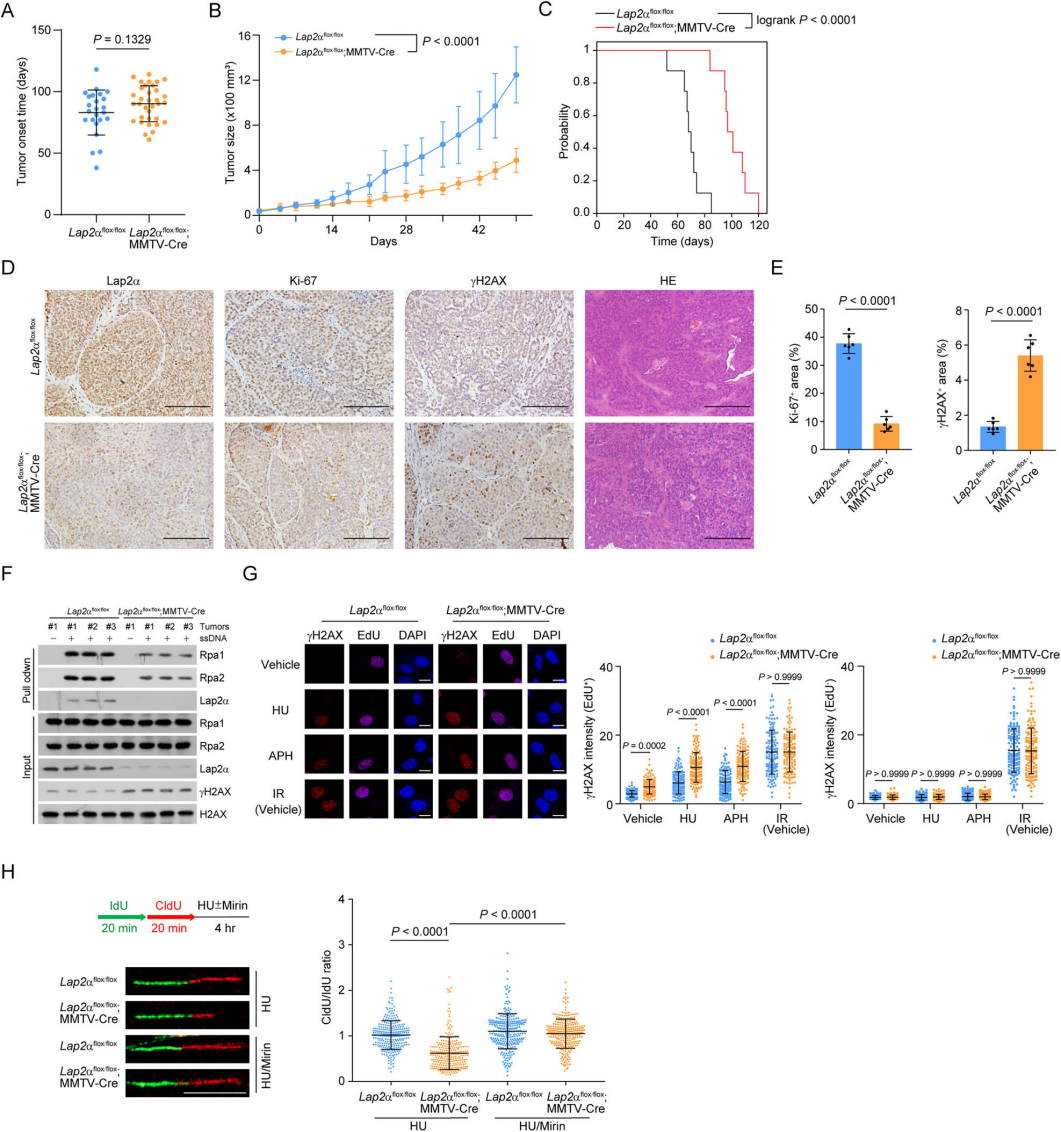
Lap2α acts intrinsically in promoting mammary gland tumor progression. **A** Tumor onset date of *Lap2*α^flox/flox^ and *Lap2*α^flox/flox^;MMTV-Cre mice. Each point represents an individual animal (*n* = 24; *n* = 34). **B** Mammary gland tumor growth of *Lap2*α^flox/flox^ or *Lap2*α^flox/flox^;MMTV-Cre mice (*n* = 8). Tumor size was monitored and shown. **C** Kaplan–Meier overall survival analysis of *Lap2*α^flox/flox^ and *Lap2*α^flox/flox^;MMTV-Cre mice bearing PyMT tumors (*n* = 8). **D** IHC analysis of Lap2*α*, Ki-67 and γH2AX in PyMT-tumors from *Lap2α*^flox/flox^ and *Lap2α*^flox/flox^;MMTV-Cre mice. Representative images are shown. **E** The expression level of Lap2*α*, Ki-67, and γH2AX from IHC stainings was scored according to the staining intensity and extent (*n* = 6). **F** ssDNA-pull down assays with nuclear extracts of PyMT-tumor cells from *Lap2α*^flox/flox^ or *Lap2α*^flox/flox^;MMTV-Cre mice. **G** Immunostaining and confocal microscopy analysis of γH2AX foci formation in PyMT-tumor cells from *Lap2α*^flox/flox^ or *Lap2α*^flox/flox^;MMTV-Cre mice under HU (2 mM, 8 h), APH (0.5 M, 8 h), or 2 h post-IR (4 Gy) treatment (*n* > 100 from two independent experiments). **H** DNA fiber assay with PyMT-tumor cells of *Lap2α*^flox/flox^ or *Lap2α*^flox/flox^*;*MMTV-Cre mice. Cells were labeled with IdU and CldU for the indicated time followed by HU treatment in the absence or presence of MRE11 inhibitor. The ratios of CldU and IdU length were calculated in each treatment (*n* > 100 from two independent experiments). Data are mean ± SDs for (**A**), (**B**), (**E**), (**G**) and (**H**); Statistical tests were performed by Welch’s *t* test (**A**, **E**), two-way ANOVA with Tukey’s multiple comparisons test (**B**), log-rank test (**C**) and Kruskal–Wallis test with Dunn’s multiple comparisons test (**G**) and (**H**). Scale bar: 100 m for (**D**) and 10 m for (**G**) and (**H**).

To further confirm that Lap2α acts autonomously in mammary gland tumor cells, MMTV-Cre mice were crossed with *Lap2*α^flox/flox^ mice to specifically deplete Lap2α in mammary gland epithelium. The development of mammary tumors in *Lap2*α^flox/flox^ and *Lap2*α^flox/flox^;MMTV-Cre mice was monitored. We found that the incidence and latency of tumor development were comparable between the two mouse groups (Fig. 4A), whereas the tumors in *Lap2*α^flox/flox^;MMTV-Cre mice grew more slowly than those in *Lap2*α^flox/flox^ mice (Fig. 4B). Survival analysis revealed that *Lap2*α knockout in epithelial cells improved the lifespan of tumor-bearing mice (Fig. 4C). These findings suggest that Lap2α promotes mammary gland tumor progression rather than mammary gland tumor initiation.

Immunohistochemical (IHC) analysis showed that *Lap2*α knockout led to decreased Ki67 levels and an upregulation of H2AX in epithelial tumor cells (Fig. 4D, E). Cultured tumor cells from *Lap2*α^flox/flox^;MMTV-Cre mice exhibited reduced RPA loading efficiency and increased DNA damage (Fig. 4F). Specifically, the enhanced H2AX signal was correlated with the S-phase in *Lap2*α knockout tumor cells treated with HU or APH, whereas no such correlation was found following IR exposure (Fig. 4G). Additionally, DNA fiber assays using nucleoside analogs 5’-iodo-2-deoxyuridine (IdU) and 5’-chloro-2-deoxyuridine (CldU) sequentially labeled ongoing replication tracts, followed by HU treatment, revealed that the CldU/IdU ratios were significantly reduced in HU-treated *Lap2*α knockout tumor cells, implying a defect in stalled fork protection (Fig. 4H). This effect was suppressed by mirin, an inhibitor of the DNA end resection factor MRE11 [30] (Fig. 4H), further indicating that LAP2α protects stalled replication forks from degradation by facilitating RPA deposition in tumor cells. Collectively, these results suggest that LAP2α-mediated mammary gland tumor progression is, in part, facilitated by promoting RPA deposition and counteracting replication stress-associated DNA damage in tumor cells.

### LAP2α deficiency sensitizes breast tumors to chemotherapeutic drugs

**Fig. 5:**
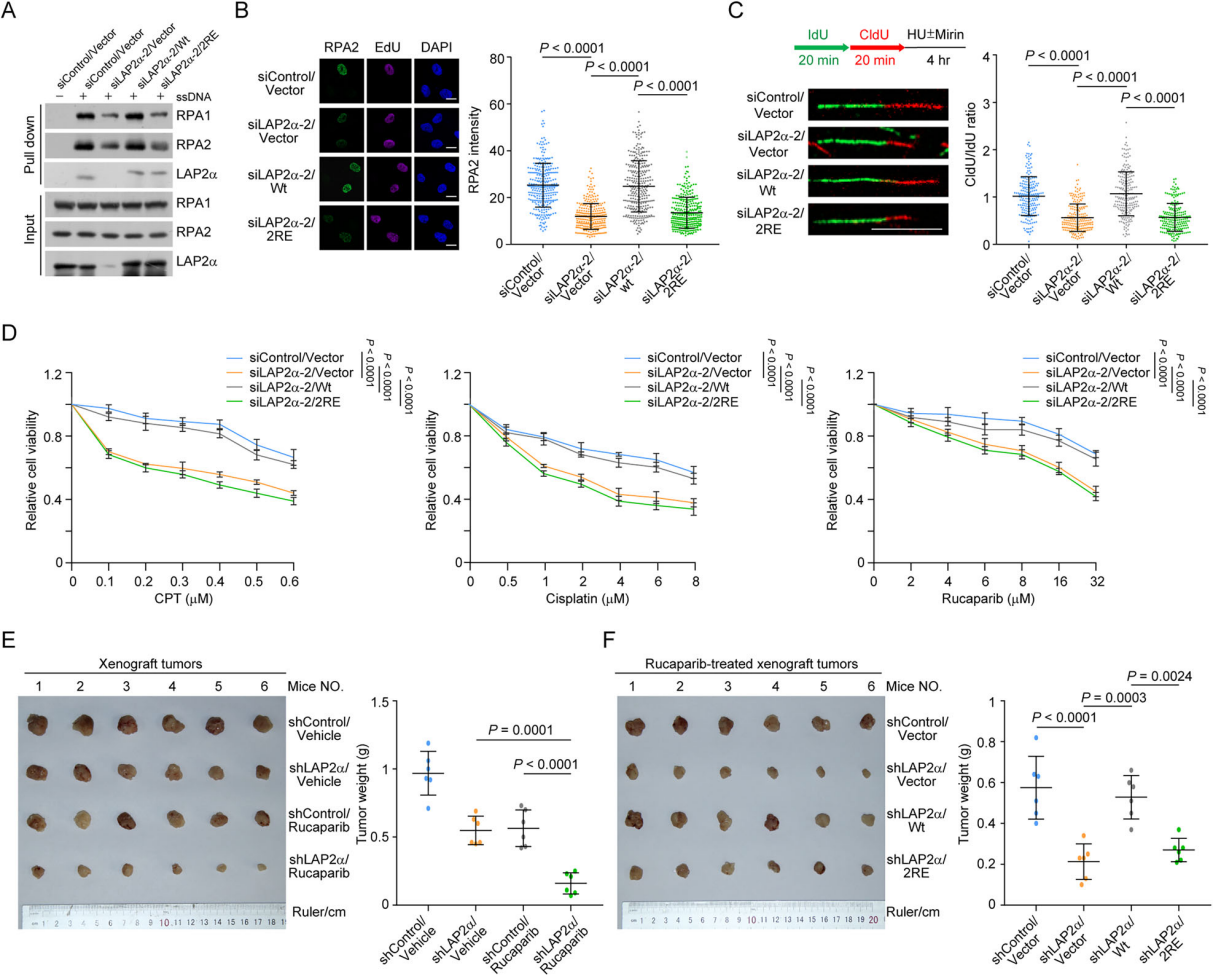
LAP2*α* deficiency sensitizes breast tumors to chemotherapeutic drugs. **A** ssDNA-pull down assays with nuclear extracts from MDA-MB-231 cells. LAP2*α*/Wt or LAP2*α*/2RE stably expressing MDA-MB-231 cells were transfected with control siRNA or LAP2*α* 3’UTR siRNA before collection. **B** Immunostaining and confocal microscopy analysis of RPA2 foci formation in MDA-MB-231 cells used in (**A**) under HU treatment (*n* > 150 from biological triplicate experiments). **C** DNA fiber assay with MDA-MB-231 cells expressing LAP2*α* 3’UTR siRNA and LAP2*α*/Wt or LAP2*α*/2RE. Cells were labeled with IdU and CldU for 20 min followed by HU treatment. The ratios of CldU and IdU length were calculated in each treatment (*n* > 100 from two independent experiments). **D** Survival analysis of MDA-MB-231 cells expressing LAP2*α* 3’UTR siRNA and LAP2*α*/Wt or LAP2α/2RE under different drug treatments (*n* = 3 from biological triplicate experiments). **E** Tumor weight of xenografts from control or LAP2*α*-knockdown MDA-MB-231 cells. SCID mice carrying tumors were treated with vehicle or rucaparib every two days (*n* = 6). **F** Tumor weight of xenografts from LAP2*α*-knockdown MDA-MB-231 cells expressing LAP2*α*/Wt or LAP2*α*/2RE. SCID mice carrying tumors were treated with vehicle or rucaparib every 2 days (*n* = 6). Data are mean ± SDs for (**B**–**F**). Statistical tests were performed by Kruskal–Wallis test with Dunn’s multiple comparisons test (**B**, **C**), two-way ANOVA with Tukey’s multiple comparisons test (**D**) and one-way ANOVA with Tukey’s multiple comparisons test (**E**, **F**). Scale bar: 10 μm for (**B**) and (**C**).

To further investigate the role of LAP2α in breast tumorigenesis, distinct LAP2α siRNAs were transfected into breast cancer cells from different molecular subtypes. Survival analysis showed that LAP2α depletion sensitized these cells to camptothecin (CPT), cisplatin, and rucaparib (Supplementary Fig. S3A–S3C). We then generated MDA-MB-231 cells stably expressing wild-type LAP2α (LAP2α/Wt) and its variant LAP2α/2RE (R86E and R88E), which fails to interact with RPA and promote RPA deposition as previously characterized [31]. Both ssDNA pull-down and immunostaining followed by confocal microscopy, revealed that LAP2α/2RE was not able to rescue LAP2α depletion-associated defects in RPA loading (Fig. 5A, B). Similar results were obtained when replication fork stability and cellular fitness were examined in these cells (Fig. 5C, D). These results suggest that LAP2α-promoted drug resistance largely depends on LAP2α-mediated RPA deposition and genome surveillance.

We next orthotopically transplanted wild-type and LAP2α stably knockdown MDA-MB-231 breast cancer cells into the mammary fat pads of 6-week-old immunocompromised severe combined immunodeficiency (SCID) female mice. The results showed that both LAP2α knockdown and rucaparib treatment suppressed tumor growth, with the combination therapy providing greater therapeutic benefit than either treatment alone (Fig. 5E). Consistently, LAP2α-depleted tumors exhibited an increased level of γH2AX and reduced Ki-67 expression (Supplementary Fig. S3D), further indicating a synergistic effect between LAP2α deficiency and genotoxic insults. We then stably integrated shRNA-resistant LAP2α/Wt and LAP2α/2RE into LAP2α-depleted MDA-MB-231 cells and assessed tumor formation in a xenograft model. The results revealed that LAP2α/Wt, but not LAP2α/2RE, could rescue the synthetically lethal effects associated with LAP2α deficiency (Fig. 5F and Supplementary Fig. S3E). LAP2α has been previously reported to implicate in telomere maintenance [32] and in the accumulation of 53BP1 [33]. The LAP2α/2RE mutant retains normal activity in 53BP1 recruitment and telomere maintenance, ruling out·the possibility that those processes are involved in LAP2α/2RE-induced genome instability and tumor vulnerability (Fig. 5B and Supplementary Fig. S3F, S3G). Collectively, these findings support the notion that LAP2α-promoted RPA loading plays a critical role in protecting tumor cells from DNA damage, and that targeting LAP2α or disrupting the LAP2α-RPA interaction may offer a promising therapeutic strategy for breast cancer treatment.

### LAP2α directly stimulates the loading of RPA onto ssDNA

**Fig. 6:**
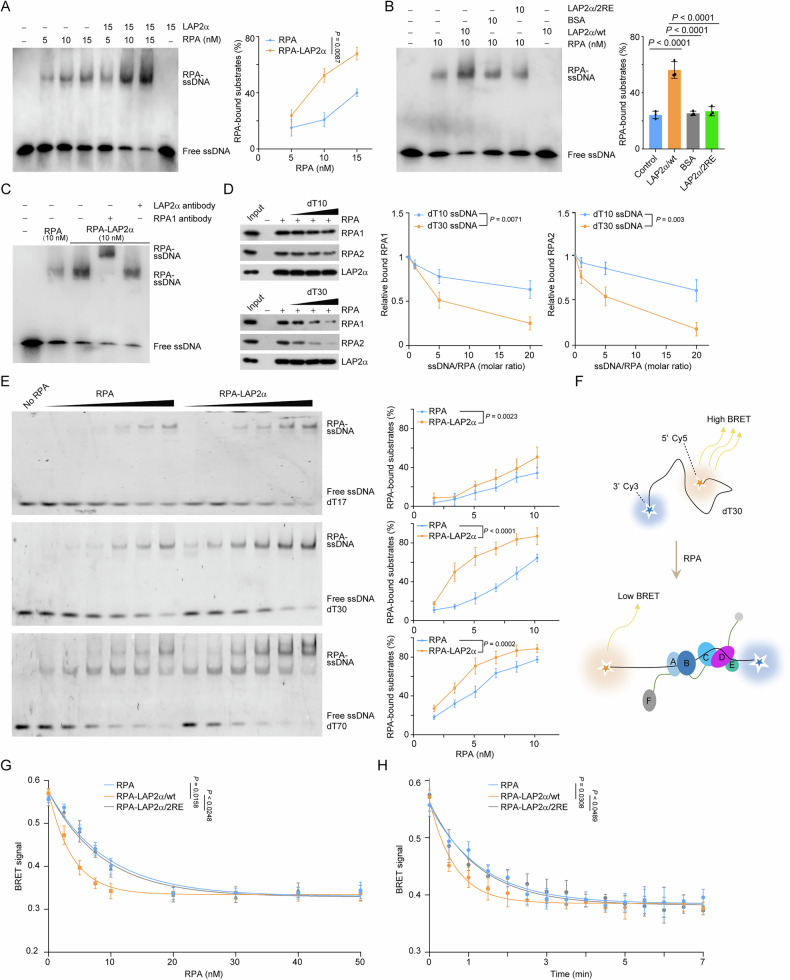
LAP2*α* directly stimulates the loading of RPA onto ssDNA. **A** Analysis of ssDNA-RPA binding in the absence or presence of LAP2*α*. EMSAs were performed with 5’ biotin-labeled 34-nt ssDNA (10 nM) and an increasing amount of RPA in the absence or presence of LAP2*α* followed by electrophoresis and visualization. Free and bound DNA is marked as indicated. The proportion of RPA-bound ssDNA was quantified. **B** Analysis of ssDNA-RPA binding in the presence of LAP2*α*/Wt or LAP2*α*/2RE. EMSAs analogous to (**A**) were performed. Free and bound DNA is marked as indicated. The proportion of RPA-bound ssDNA was quantified. **C** Analysis of the composition of ssDNA-RPA complex in the presence of LAP2*α*. Antibodies against RPA1 or LAP2*α* were added and EMSAs analogous to (**B**) were performed. **D** LAP2*α*-RPA pull-down with oligonucleotide titrations. RPA was incubated with immobilized recombinant LAP2*α*, and the preformed complexes were challenged with 1-, 5- and 20-fold molar excesses of either dT10 or dT30 ssDNA. The remaining proteins bound to the beads were examined by immunoblotting and quantified as shown. **E** The stimulatory effect of LAP2*α* on ssDNA-RPA binding with shorter and longer stretches of oligo(dT). EMSAs were performed with 5’ Cy3-labeled dT17, dT30, and dT70 ssDNA (5 nM) and an increasing amount of RPA in the absence or presence of LAP2*α* (10 nM) followed by electrophoresis and visualization. Free and bound DNA is marked as indicated. The proportion of RPA-bound ssDNA was quantified. **F** An illustration of how the RPA-BRET system works. Free dT30 ssDNA yields a high BRET signal due to the close proximity of the DNA ends labeled by 3’-Cy3 (BRET donor) and 5’-Cy5 (BRET acceptor), while binding of RPA straightens the ssDNA thereby increasing the Cy3-Cy5 distance and weakening BRET signal. **G** BRET analysis of the configuration of ssDNA when RPA bound in the absence or presence of LAP2*α* variants. Indicated concentrations of RPA-LAP2*α*/Wt or RPA-LAP2*α*/2RE were added to the solution containing 10 nM dT30 oligo decorated with the Cy3 and Cy5 dye. BRET signal was monitored after 10 minutes of incubation. **H** BRET analysis of the binding kinetics of ssDNA-RPA in the absence or presence of LAP2*α* variants. RPA-LAP2*α*/Wt or RPA-LAP2*α*/2RE were incubated with Cy3- and Cy5-labeled dT30 oligo (10 nM for each). The kinetics of BRET signal was monitored. Data are mean ± SDs for (**A**), (**B**), (**D**), (**E**), (**G**) and (**H**) from biological triplicate experiments. Statistical tests were performed by two-way ANOVA with Tukey’s multiple comparisons test (**A**, **D**, **E**, **G**, **H**), and one-way ANOVA with Tukey’s multiple comparisons test (**B**).

To determine whether LAP2α actively facilitates RPA loading onto ssDNA, we analyzed RPA binding to ssDNA using an electrophoretic mobility shift assay (EMSA) in the presence or absence of LAP2α. First, increasing amounts of RPA were incubated with a fixed amount of biotin-labeled 34-nucleotide (nt) ssDNA, and the resulting DNA-protein complexes were separated from unbound DNA using non-denaturing gel electrophoresis. The relative abundance of free and bound DNA was then detected using chemiluminescent imaging. Preincubation of LAP2α with RPA significantly enhanced RPA binding efficiency to ssDNA in an RPA dose-dependent manner (Fig. 6A), whereas LAP2α alone did not bind to ssDNA (Fig. 6A). In contrast, LAP2α/2RE, which cannot bind RPA, failed to promote ssDNA-RPA complex formation (Fig. 6B). These results indicate that LAP2α actively promotes the formation of ssDNA-RPA complexes in vitro through its interaction with RPA (Supplementary Fig. S4A).

To further analyze the composition of the ssDNA-RPA complex, we conducted an EMSA assay using antibodies against RPA1 and LAP2α. A supershift band was observed upon the addition of anti-RPA1, whereas anti-LAP2α did not produce any detectable supershift (Fig. 6C). These findings suggest that RPA binding to ssDNA may disrupt its interaction with LAP2α. To investigate this further, pre-formed LAP2α-RPA complexes immobilized on beads were exposed to increasing concentrations of oligodeoxythymidine (oligo(dT)). We observed that addition of a short ssDNA stretch, consisting of a 10-nt oligo(dT) (dT10), caused a gradual dissociation of RPA from LAP2α (Fig. 6D). Furthermore, titration with a longer ssDNA fragment (dT30) resulted in a more efficient dissociation of RPA from LAP2α (Fig. 6D). This is likely due to a conformational change in RPA, transitioning from an 8-10 nt to a 30-nt binding mode, which may facilitate the formation of the ssDNA-RPA complex and lead to the disassembly of the LAP2α-RPA complex. These results suggest that LAP2α is not part of the final ssDNA-RPA complex, and that LAP2α functions as a chaperone in RPA loading (Supplementary Fig. S4B).

LAP2α was then titrated over a range of 1–50 nM in the presence of 5 nM RPA and 5’ Cy3-labeled dT30 ssDNA, and fluorescence-based EMSA assays revealed that LAP2α facilitated RPA binding to ssDNA in a near-stoichiometric manner rather than a catalytic role, while LAP2α/2RE, which cannot bind RPA, failed to promote ssDNA-RPA complex formation (Supplementary Fig. S4C). This observation aligns with the longer turnover rate of the LAP2α-RPA binding process [31]. These findings suggest that it is likely that LAP2α requires time to deliver RPA and optimize its positioning or repositioning on ssDNA. To investigate how LAP2α influences the formation of ssDNA-RPA complexes, we evaluated its stimulatory effect on ssDNA-RPA binding using Cy3-labeled shorter and longer stretches of oligo(dT). Normalization against RPA binding to the corresponding oligo(dT) lengths revealed that LAP2α stimulated RPA binding to all forms of ssDNA, albeit to varying extents (Fig. 6E). Notably, the stimulatory effect was less pronounced for dT17 compared to dT30 (Fig. 6E), further suggesting that LAP2α facilitates the transition of ssDNA-RPA binding by promoting RPA to adopt an extended conformation. However, no cooperative activity was observed in LAP2α-mediated RPA loading, as no additional stimulatory effect was detected for dT70 (Fig. 6E).

Next, we employed BRET-based analysis to examine RPA’s ability to stretch ssDNA. In this assay, dT30 was labeled at both termini with Cy3 (BRET donor) and Cy5 (BRET acceptor) fluorophores (Fig. 6F). A high BRET state indicates bent ssDNA due to the close proximity of the two dyes, whereas a low BRET state reflects an extended and near-linear ssDNA conformation upon RPA binding (Fig. 6F). In the presence of LAP2α, RPA extended ssDNA more efficiently, requiring less RPA to saturate the BRET signal (Fig. 6G). Kinetic analysis revealed that LAP2α significantly shortened the saturation time for ssDNA-RPA binding, resulting in a faster stretching pattern (Fig. 6H). Notably, these effects were dependent on the LAP2α-RPA interaction, as LAP2α/2RE did not influence RPA’s stretching activity or ssDNA-RPA binding kinetics (Fig. 6F–H). To validate our in vitro findings, we have generated a doxycycline (DOX)-inducible LAP2α overexpression system in MDA-MB-231 cells. Based on this system, we then demonstrated that induced expression of LAP2α promoted RPA deposition and replication stress-associated DNA damage repair in LAP2α-depleted MDA-MB-231 cells (Supplementary Fig. S4D, S4E). Furthermore, immunoblotting analysis indicated that LAP2α depletion markedly reduced the phosphorylation level of RPA2 S33 and CHK1 S345 in MDA-MB-231 cells, while its overexpression reversed phosphorylation of these markers (Supplementary Fig. S4F). Since severe or unrepaired DNA damage triggers the intrinsic apoptosis [34, 35], and LAP2α deficiency leads to the accumulation of DNA damage, we investigated the effect of LAP2α loss on apoptosis. We quantified apoptosis rates and found that loss of LAP2α induced apoptosis in tumor cells, while its overexpression reversed the apoptosis effects associated with LAP2α deficiency (Supplementary Fig. S4G). Collectively, these findings provide additional mechanistic insight into how LAP2α promotes RPA binding to ssDNA in vitro and support the hypothesis that LAP2α functions as a chaperone for RPA loading.

## Discussion

This study reveals that LAP2α promotes the progression of breast tumors, highlighting its role in promoting tumorigenesis by resolving replication stress-associated DNA damage. Our findings not only expand the known functions of LAP2α beyond its established roles in nuclear organization and genome protection but also position the LAP2α-RPA interaction as a potential therapeutic target for tumor intervention. Using genetic and orthotopic mouse models, we demonstrate that LAP2α functions as a tumor-promoting factor, likely by aiding RPA deposition and mitigating replication stress-induced DNA damage. Moreover, we provide evidence that targeting LAP2α expression or disrupting the LAP2α-RPA interaction significantly increases tumor sensitivity to chemotherapeutic agents. Finally, in vitro experiments offer additional mechanistic insights, revealing that LAP2α directly facilitates RPA loading onto single-stranded DNA (ssDNA), further underscoring its role in genome stability.

The ssDNA-RPA complex is a key regulator of eukaryotic DNA metabolism [11], yet the mechanisms governing its formation remain incompletely understood. We provided multiple lines of evidence implicating LAP2α in regulating RPA binding to ssDNA in vitro. First, our previous study demonstrated that LAP2α directly interacts with the DBD-A domain of RPA1 [24], which binds the 5’end of ssDNA in both low- and high-affinity RPA-binding modes and undergoes dynamic association and dissociation with ssDNA. This interaction closely resembles the contacts between RPA and SV40 T-antigen or CDC45, which facilitate RPA deposition onto ssDNA during replication [36, 37]. Second, in this study, gel shift assays and BRET assays revealed that LAP2α enhances both the binding efficiency and ssDNA-stretching ability of RPA. Although LAP2α interacts with chromatin and double-stranded DNA (dsDNA), gel shift assays confirmed that it does not directly bind ssDNA, suggesting that its role in RPA loading is not mediated by DNA bending or stretching. Third, we demonstrated that the LAP2α facilitates the formation of the ssDNA-RPA complex via interaction with RPA, as the LAP2α/2RE mutant, which is incapable of binding RPA, failed to facilitate RPA loading. Fourth, binding titration experiments using ssDNA of varying lengths indicated that LAP2α promotes a transition in RPA binding modes, likely by enhancing the engagement of additional OB-fold domains of RPA to occupy longer ssDNA stretches. This transition may be driven by an allosteric effect of LAP2α on the DBD-A domain, inducing conformational changes in other DBDs to efficiently complete RPA loading. Finally, in contrast to CDC45 [36], LAP2α facilitates RPA loading in a near-stoichiometric manner rather than acting catalytically.

LAP2α has been previously reported to function as a tumor suppressor by inhibiting the transcriptional activity of E2F through its interaction with pRb and lamin A/C [38]. However, this hypothesis lacks substantial experimental evidence. In this study, we found that LAP2α expression is significantly elevated in breast cancer, irrespective of the histological or molecular subtypes of the tumor tissues. Notably, genetic and xenograft mouse models revealed that LAP2α deficiency led to the accumulation of DNA damage and markedly inhibited breast cancer progression once the tumor developed. Our observations indicate that fork stability, as well as RPA loading capacity, were severely impaired in mammary gland tumors from *Lap2*α-knockout mice and in cultured human cancer cell lines. These findings suggest that, during tumor progression, particularly in breast carcinoma, LAP2α’s role in genome surveillance may override its transcriptional repression and cell cycle-suppressing functions. Tumor cells typically exhibit elevated endogenous replication stress, which requires RPA-mediated fork protection, ATR activation, and HR repair for resolution [39]. Therefore, it is reasonable to see that LAP2α-promoted RPA loading contributes to breast tumorigenesis. However, other changes induced by LAP2α deficiency should not be overlooked. For instance, its elevated expression is frequently associated with the upregulation of tumor-relevant signaling pathways [23]. Additionally, LAP2α overexpression is possibly triggered by c-Myc amplification or p53 inactivation [20]. Future studies will be needed to further investigate the mechanisms that regulate LAP2α expression at the transcriptional or co-transcriptional splicing levels.

Given that LAP2α is required for maintaining genome stability by promoting RPA loading, we hypothesized that modulation of RPA availability on ssDNA by targeting LAP2α could influence the therapeutic efficacy of chemotherapeutic drugs. Indeed, we demonstrated that LAP2α-deficient tumors were more sensitive to rucaparib and cisplatin. Rescue experiments using a LAP2α mutant, which is defective in binding and loading RPA, further corroborated the idea that failure to efficiently deposit RPA contributes to this synthetically lethal effect. Although it remains to be tested whether this phenomenon applies to other cancers with high LAP2α expression, our findings suggest that modulating the occupancy of RPA on ssDNA by targeting LAP2α or disrupting LAP2α-RPA binding may serve as a promising strategy for breast cancer intervention. Furthermore, LAP2α could potentially be used as a prognostic marker for breast cancer, and its expression might be clinically useful for predicting therapeutic outcomes with platinum-based drugs or PARP inhibitors (PARPis). Our findings are primarily derived from preclinical models, and the clinical significance of LAP2α in human breast cancer requires validation through large-scale patient studies. Furthermore, the development of specific LAP2α-RPA inhibitors will be essential for translating these findings into clinical applications.

## Supplementary information

Supplemental Figures

Table S1

Full and uncropped western blots

## Data Availability

All relevant data are available from the authors on request. Full and uncropped western blots are in the supplemental materials.
