# Supplementary material for: LAP2α drives breast tumorigenesis by mitigating replication stress
Source: Cell Death Dis. 2026 Feb 3;17(1):201. doi: 10.1038/s41419-026-08433-6 (PMC12894886; doi:10.1038/s41419-026-08433-6)
Supplement: Supplementary file 1 — Supplemental Figures [file 41419_2026_8433_MOESM1_ESM.pdf]

Figure S1

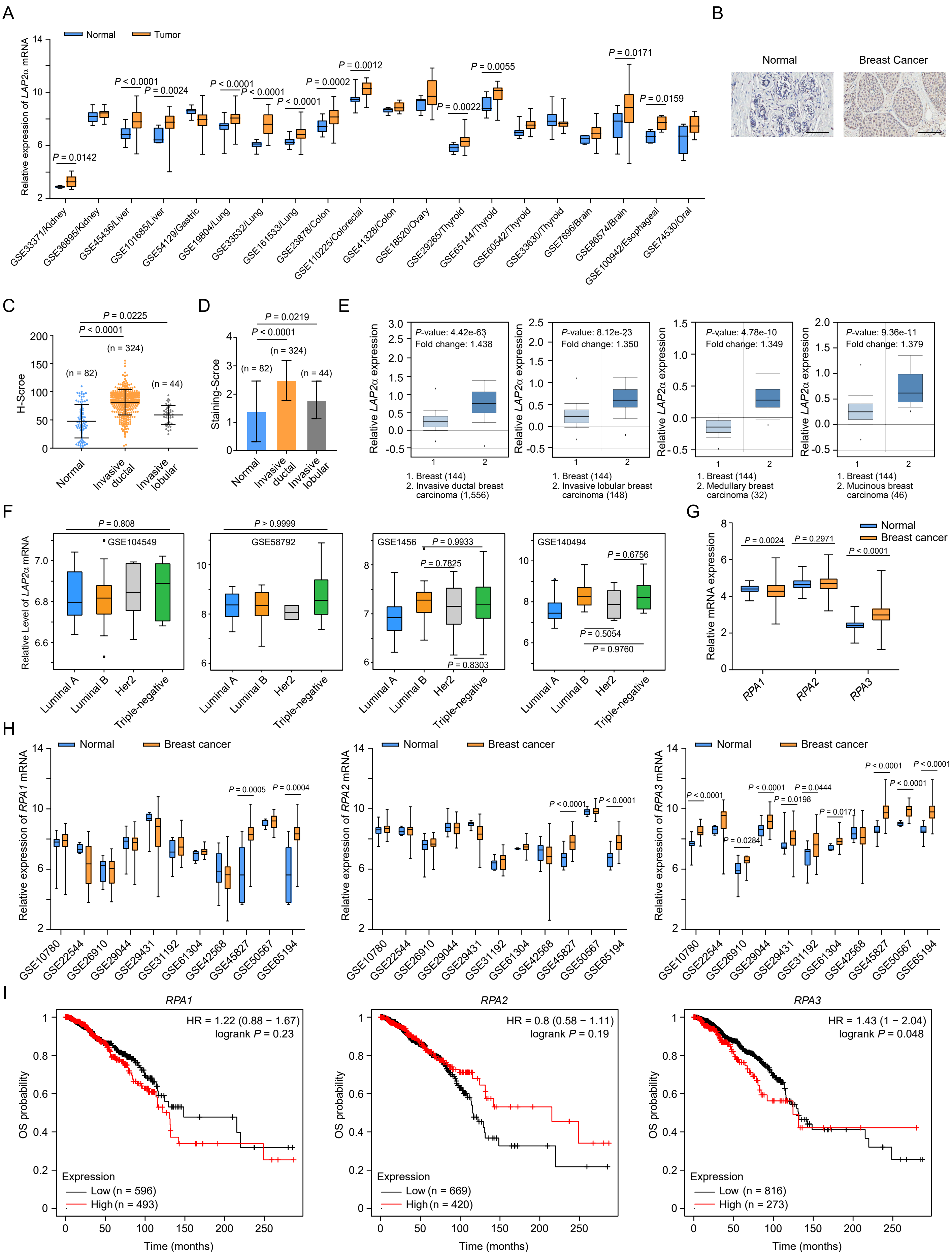
A
Normal
Tumor
Relative expression of LAP2α mRNA
P = 0.0142
P < 0.0001
P = 0.0024
P < 0.0001
P < 0.0001
P < 0.0001
P = 0.0002
P = 0.0012
P = 0.0022
P = 0.0055
P = 0.0171
P = 0.0159
GSE33371/Kidney
GSE36895/Kidney
GSE45436/Liver
GSE101685/Liver
GSE54129/Gastric
GSE19804/Lung
GSE33532/Lung
GSE161533/Lung
GSE23878/Colon
GSE110225/Colorectal
GSE41328/Colon
GSE18520/Ovary
GSE29265/Thyroid
GSE65144/Thyroid
GSE60542/Thyroid
GSE33630/Thyroid
GSE7696/Brain
GSE86574/Brain
GSE100942/Esophageal
GSE74530/Oral
B
Normal
Breast Cancer
C
H-Scroe
P = 0.0225
P < 0.0001
(n = 82)
(n = 324)
(n = 44)
Normal
Invasive ductal
Invasive lobular
D
Staining-Scroe
P = 0.0219
P < 0.0001
(n = 82)
(n = 324)
(n = 44)
Normal
Invasive ductal
Invasive lobular
E
Relative LAP2α expression
P-value: 4.42e-63
Fold change: 1.438
1. Breast (144)
2. Invasive ductal breast carcinoma (1,556)
P-value: 8.12e-23
Fold change: 1.350
1. Breast (144)
2. Invasive lobular breast carcinoma (148)
P-value: 4.78e-10
Fold change: 1.349
1. Breast (144)
2. Medullary breast carcinoma (32)
P-value: 9.36e-11
Fold change: 1.379
1. Breast (144)
2. Mucinous breast carcinoma (46)
F
Relative Level of LAP2α mRNA
P = 0.808
GSE104549
P > 0.9999
GSE58792
GSE1456
P = 0.9933
P = 0.7825
P = 0.8303
GSE140494
P = 0.6756
P = 0.5054
P = 0.9760
Luminal A
Luminal B
Her2
Triple-negative
G
Relative mRNA expression
Normal
Breast cancer
P = 0.0024
P = 0.2971
P < 0.0001
RPA1
RPA2
RPA3
H
Normal
Breast cancer
Relative expression of RPA1 mRNA
P = 0.0005
P = 0.0004
Relative expression of RPA2 mRNA
P < 0.0001
P < 0.0001
Relative expression of RPA3 mRNA
P < 0.0001
P = 0.0284
P < 0.0001
P = 0.0198
P = 0.0444
P = 0.0171
P < 0.0001
P < 0.0001
P < 0.0001
GSE10780
GSE22544
GSE26910
GSE29044
GSE29431
GSE31192
GSE61304
GSE42568
GSE45827
GSE50567
GSE65194
I
RPA1
HR = 1.22 (0.88 − 1.67)
logrank P = 0.23
Expression
Low (n = 596)
High (n = 493)
RPA2
HR = 0.8 (0.58 − 1.11)
logrank P = 0.19
Expression
Low (n = 669)
High (n = 420)
RPA3
HR = 1.43 (1 − 2.04)
logrank P = 0.048
Expression
Low (n = 816)
High (n = 273)
OS probability
Time (months)

Figure S1 (Figure 1 Continued). LAP2α is implicated in breast tumorigenesis. (A) Analysis of the expression of *LAP2α* in different types of cancers and the corresponding normal tissues from multiple independent data of Gene Expression Omnibus (GEO) with *LAP2α* (NM_003276) specific probe 203432_at. *P value* with Welch's t test for *LAP2α* highly expressed datasets. (B) Representative images of normal and breast cancer samples that corresponds to Figure 1B. (C) Quantitative analysis of the expression level of LAP2α in different histological subtypes of breast cancer samples and normal breast tissues according to IHC stainings. *P value* with one-way ANOVA with Dunnett's multiple comparisons test. (D) Staining score corresponds to the H-Score presented in (C). (E) Analysis of the expression of *LAP2α* in distinct histological subtypes of breast cancer samples and normal breast tissues from Curtis Breast of Oncomine with *LAP2α* specific probe. (F) Analysis of the expression of *LAP2α* in distinct molecular subtypes of breast cancer samples from GEO datasets with *LAP2α* specific probe. (G) Analysis of the expression of *RPA1*, *RPA*2, and *RPA3* in breast cancer samples and normal breast tissues from TCGA datasets. *P value* with Welch's t test. (H) Analysis of the expression of *RPA1*, *RPA*2, and *RPA3* in breast cancer samples and normal breast tissues from multiple independent GEO datasets that were used to analyze *LAP2α* expression in Figure 1A. *P value* with Welch's t test for *LAP2α* highly expressed datasets. (I) Overall survival (OS) analysis of breast cancer patients with different expression status of *RPA1*, *RPA*2, and *RPA3* with survival packages from KM plotter. Scale bar, 100 μm for (B).

Figure S2

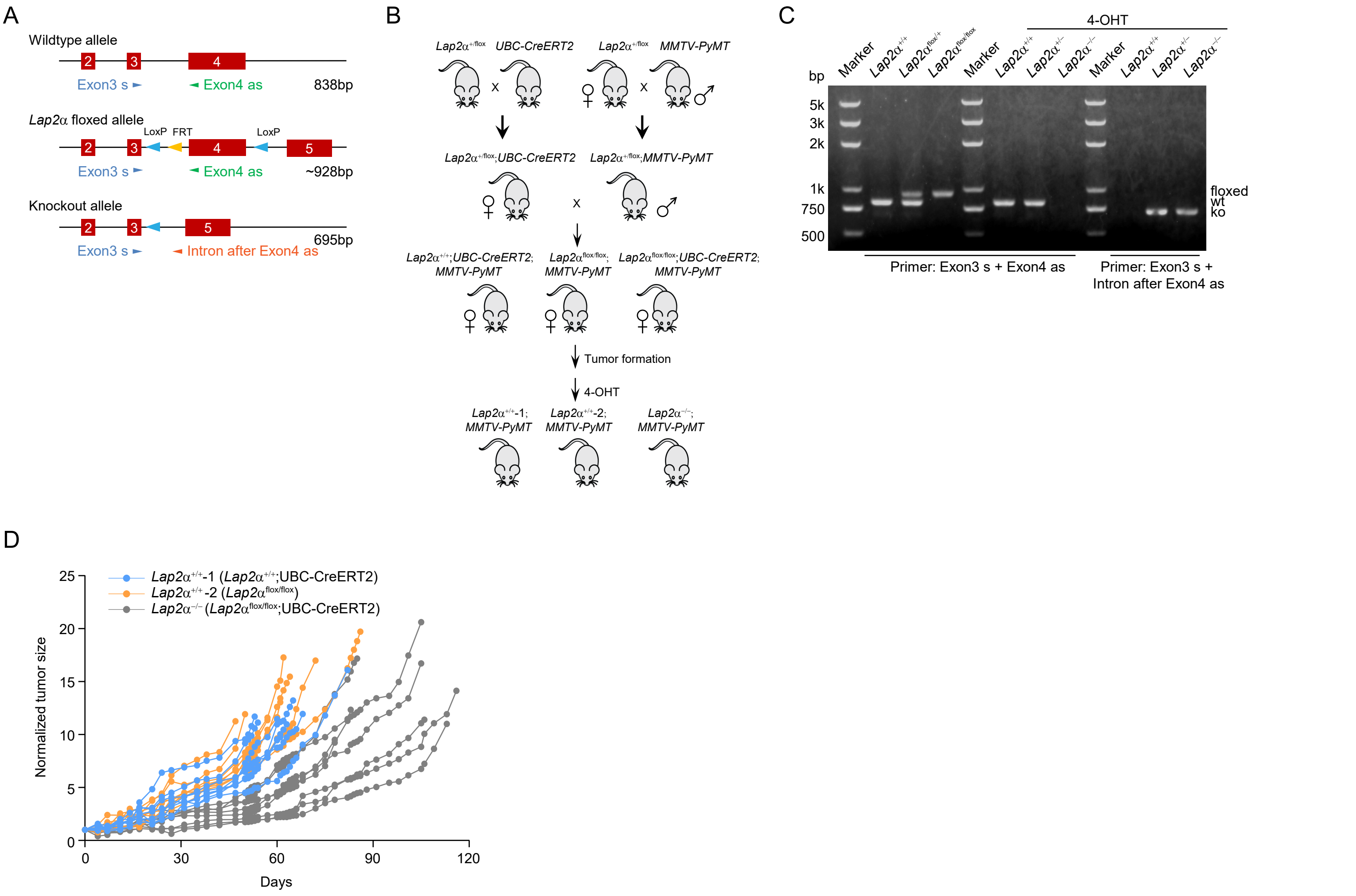

Figure S2 (Figure 2 Continued). Lap2α knockout suppresses the growth of mammary gland tumors. (A) Schematic representation of the wild type allele, floxed allele, and knockout allele the *of Lap2α* gene locus. FRT (yellow triangles) and loxP (blue triangles) recombination sites, and exons (red boxes) are shown. (B) Schematic representation of the UBC-Cre driven mouse work. (C) PCR analysis of genomic DNA prepared from tail biopsies of wild-type (*Lap2α*$^{+/+}$), heterozygous floxed (*Lap2α*$^{flox/+}$), homozygous floxed (*Lap2α*$^{flox/flox}$), heterozygous (*Lap2α*$^{+/-}$) and homozygous knockout (*Lap2α*$^{-/-}$) mice. Positions of primers reflecting wild type (wt), floxed, and knockout (ko) genotypes are indicated. Wild-type (WT), homozygous (HO), and heterozygous (HE) alleles were amplified with primers exon3 s and exon4 as, yielding products of 838 bp (WT), 928 bp (HO), or both (HE). The knockout (KO) allele was detected using exon3 s and Intron after Exon4 as, producing a 695 bp fragment. The genotyping PCR utilized forward primer Exon3 s (5'-CAGGGAACTGAATCGAGATCCTCTAC-3') paired with either reverse primer Exon4 as (5'-CACAATCCCTAGAGGACTTCACTT-3') for wild-type, homozygous and heterozygous amplification or Intron-after-Exon4-as (5'-CTGTGACTTTGCTGGCCTTCCAGTCTA-3') for knockout allele detection. (D) Individual mouse mammary tumor growth curves corresponding to Figure 2B.

Figure S3

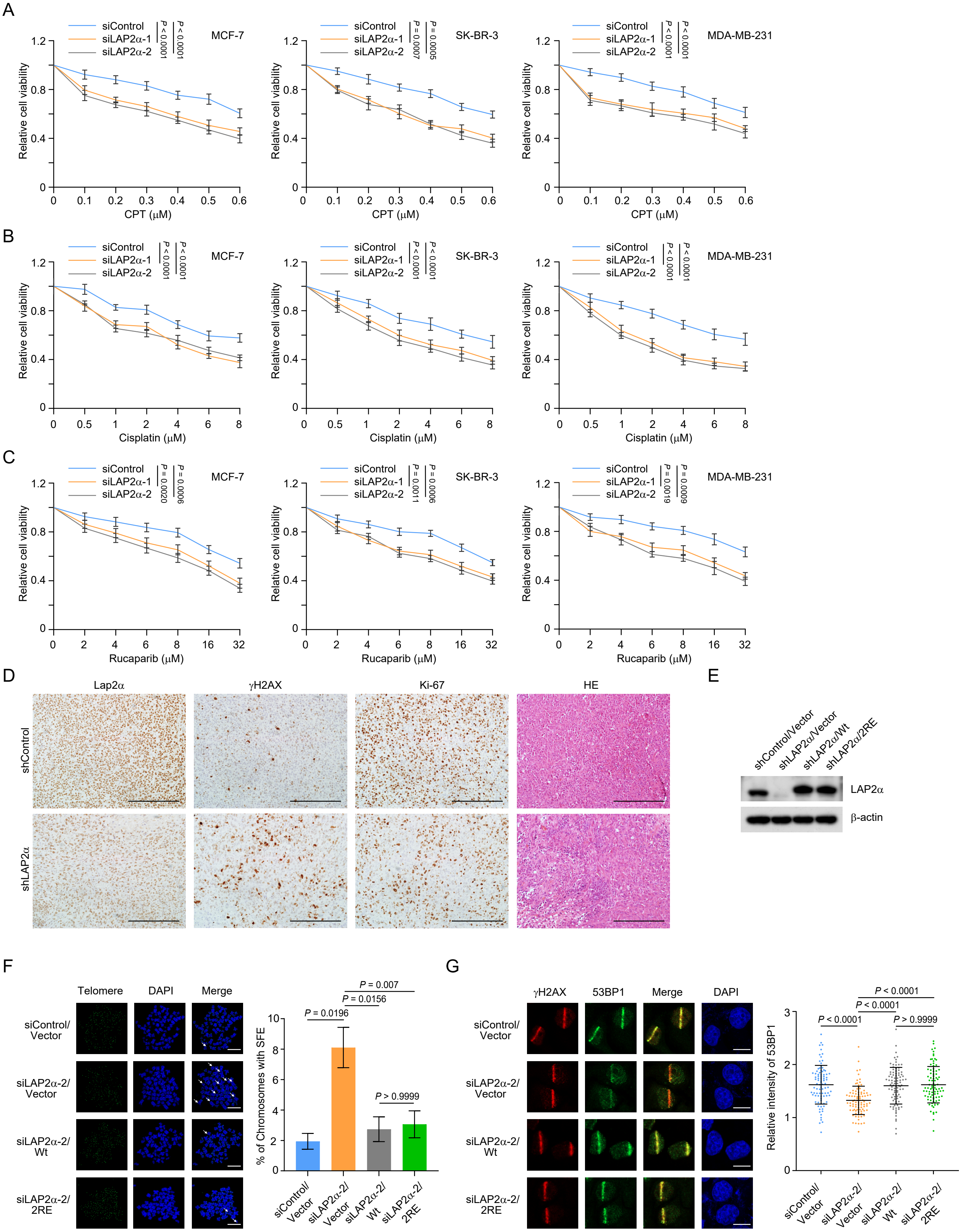

A
siControl
siLAP2α-1
siLAP2α-2
MCF-7
SK-BR-3
MDA-MB-231
Relative cell viability
CPT (μM)
B
Cisplatin (μM)
C
Rucaparib (μM)
D
Lap2α
γH2AX
Ki-67
HE
shControl
shLAP2α
E
shControl/Vector
shLAP2α/Vector
shLAP2α/Wt
shLAP2α/2RE
LAP2α
β-actin
F
Telomere
DAPI
Merge
siControl/ Vector
siLAP2α-2/ Vector
siLAP2α-2/ Wt
siLAP2α-2/ 2RE
% of Chromosomes with SFE
P = 0.007
P = 0.0156
P = 0.0196
P > 0.9999
G
γH2AX
53BP1
Merge
DAPI
Relative intensity of 53BP1
P < 0.0001
P < 0.0001
P < 0.0001
P > 0.9999

Figure S3 (**Figure 5 Continued**) **LAP2α deficiency sensitizes breast tumors to chemotherapeutic drugs.** (A) Survival analysis of MCF-7 (luminal), SK-BR-3 (her2), and MDA-MB-231 (triple-negative) cells expressing LAP2α siRNAs under CPT treatment. Data are mean ± SDs from biological triplicate experiments. *P values* were determined by two-way ANOVA followed by Tukey's multiple comparisons test. (B) Survival analysis of MCF-7, SK-BR-3, and MDA-MB-231 cells expressing LAP2α siRNAs under cisplatin treatment. Data are mean ± SDs from biological triplicate experiments. *P values* were determined by two-way ANOVA followed by Tukey's multiple comparisons test. (C) Survival analysis of MCF-7, SK-BR-3, and MDA-MB-231 cells expressing LAP2α siRNAs under rucaparib treatment. Data are mean ± SDs from biological triplicate experiments. *P values* were determined by two-way ANOVA followed by Tukey's multiple comparisons test. (D) IHC analysis of the level of LAP2α, Ki-67, and γH2AX in MDA-MB-231 tumor xenografts. Representative images are shown. (E) Immunoblotting analysis of the expression of the indicated proteins in shLAP2α cells that were stably integrated with LAP2α/Wt or LAP2α/2RE. (F) Immunostaining and confocal microscopy analysis of metaphase chromosome spreads and telomere FISH in U2OS cells expressing the indicated siRNAs and overexpressing LAP2α variants. ($n > 40$ from biological triplicate experiments). *P values* were analyzed by the Kruskal–Wallis test followed by Dunn's multiple comparisons test. (G) Quantitative analysis of 53BP1 recruitment to γH2AX-marked laser micro-irradiation sites by immunostaining and confocal microscopy in U2OS cells expressing the indicated siRNAs and overexpressing LAP2α variants ($n > 100$ from two independent experiments). *P values* were analyzed by the Kruskal–Wallis test followed by Dunn's multiple comparisons test. Scale bar: 100 μm for (D) and 10 μm for (F) and (G).

Figure S4

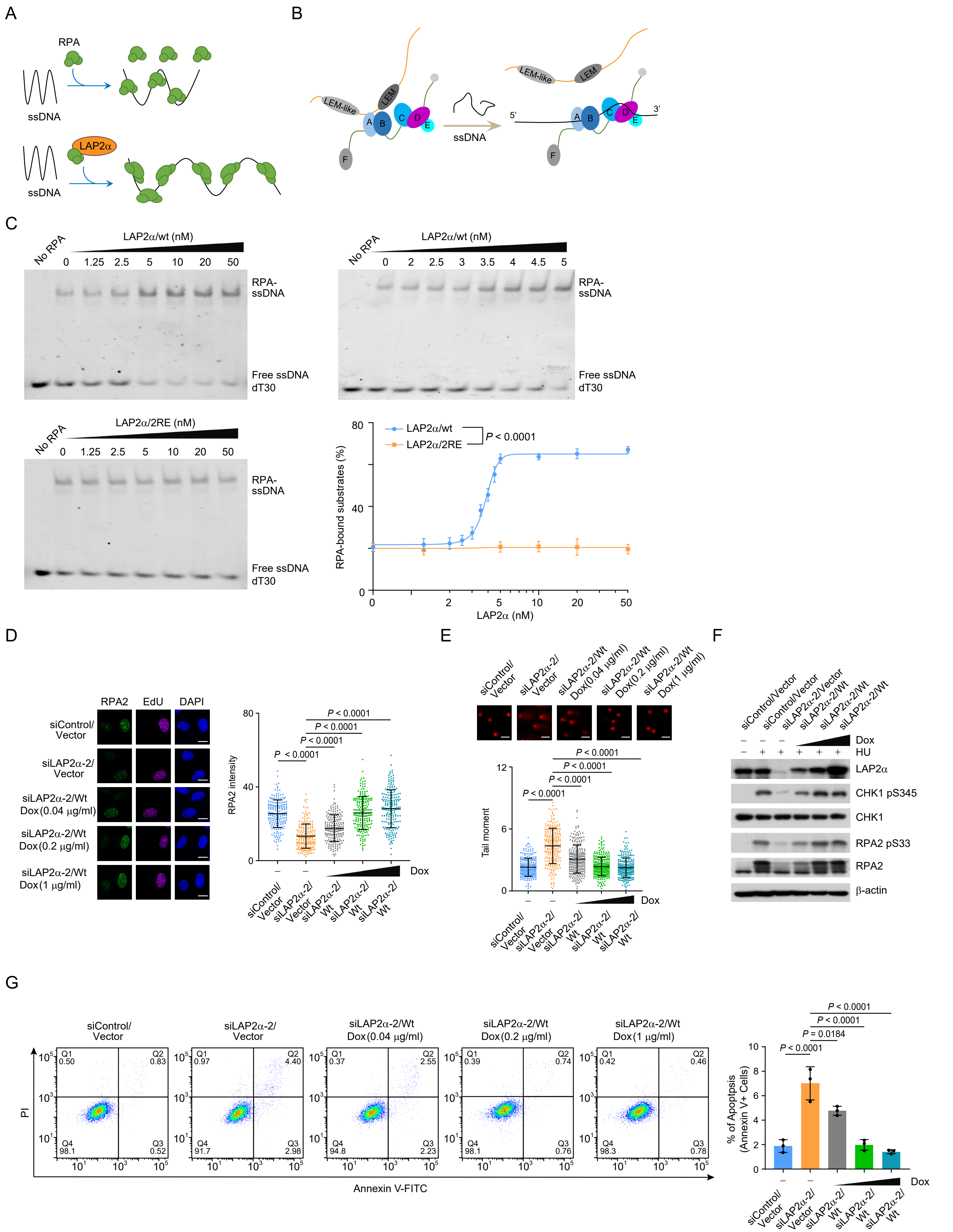

A
RPA
ssDNA
LAP2α
ssDNA
B
LEM-like
LEM
A B C D E F
ssDNA
5'
3'
C
No RPA
LAP2α/wt (nM)
0 1.25 2.5 5 10 20 50
RPA-ssDNA
Free ssDNA dT30
LAP2α/wt (nM)
0 2 2.5 3 3.5 4 4.5 5
LAP2α/2RE (nM)
0 1.25 2.5 5 10 20 50
LAP2α/wt
LAP2α/2RE
P < 0.0001
RPA-bound substrates (%)
LAP2α (nM)
D
RPA2 EdU DAPI
siControl/Vector
siLAP2α-2/Vector
siLAP2α-2/Wt Dox(0.04 μg/ml)
siLAP2α-2/Wt Dox(0.2 μg/ml)
siLAP2α-2/Wt Dox(1 μg/ml)
RPA2 intensity
P < 0.0001
Dox
E
Tail moment
P < 0.0001
Dox
F
siControl/Vector
siControl/Vector
siLAP2α-2/Vector
siLAP2α-2/Wt
siLAP2α-2/Wt
siLAP2α-2/Wt
Dox
HU
LAP2α
CHK1 pS345
CHK1
RPA2 pS33
RPA2
β-actin
G
siControl/Vector
siLAP2α-2/Vector
siLAP2α-2/Wt Dox(0.04 μg/ml)
siLAP2α-2/Wt Dox(0.2 μg/ml)
siLAP2α-2/Wt Dox(1 μg/ml)
PI
Annexin V-FITC
Q1 0.50 Q2 0.83 Q4 98.1 Q3 0.52
Q1 0.97 Q2 4.40 Q4 91.7 Q3 2.98
Q1 0.37 Q2 2.55 Q4 94.8 Q3 2.23
Q1 0.39 Q2 0.74 Q4 98.1 Q3 0.76
Q1 0.42 Q2 0.46 Q4 98.3 Q3 0.78
% of Apoptpsis (Annexin V+ Cells)
P < 0.0001
P = 0.0184
P < 0.0001
P < 0.0001
Dox

Figure S4. (**Figure 6 Continued**) **LAP2α directly stimulates the loading of RPA onto ssDNA.** (A-B) A proposed *in vitro* model of LAP2α-promoted RPA deposition on ssDNA. Structured domains of LAP2α and DBDs from A to F of RPA are indicated with ovals of distinct colour. (C) Analysis of ssDNA-RPA binding in the absence or presence of LAP2α. EMSAs were performed with 5' Cy3-labelled dT30 ssDNA (5 nM) and a fixed amount of RPA (5 nM) in the presence or absence of an increasing amount of LAP2α/Wt or LAP2α/2RE as indicated followed by electrophoresis and visualization. Free and bound DNA is marked as indicated. The proportion of RPA-bound ssDNA was quantified. Data are mean ± SDs from biological triplicate experiments. *P values* were determined by two-way ANOVA followed by Tukey's multiple comparisons test. (D) Immunostaining and confocal microscopy analysis of RPA2 foci formation in cells under HU treatment (2 mM, 4 h). Cells were transfected with control siRNA or LAP2α 3'UTR siRNA and treated with 0.04, 0.2, and 1 μg/ml doxycycline for the induction of LAP2α expression (n > 100 from two independent experiments). *P values* were analyzed by the Kruskal–Wallis test followed by Dunn's multiple comparisons test. (E) DNA damage accumulation was assessed by alkaline comet assay in the same cells as in (D) following HU (2 mM, 12 h) treatment and 12 h post-release. (n > 100 from two independent experiments). *P values* were determined by one-way ANOVA with Dunnett's multiple comparisons test. (F) Assessment of checkpoint activity by immunoblotting in cells derived from (D) in the absence or presence of HU (2 mM, 4 h). The cellular extracts were collected to examine HU-induced phosphorylation events. (G) The percentage of apoptotic cells was determined by flow cytometry in the cells shown in (D). *P values* were analyzed by the Kruskal–Wallis test followed by Dunn's multiple comparisons test. Scale bar: 10 μm for (D), and 100 μm for (E).
