# Supplementary material for: LAP2α drives breast tumorigenesis by mitigating replication stress
Source: Cell Death Dis. 2026 Feb 3;17(1):201. doi: 10.1038/s41419-026-08433-6 (PMC12894886; doi:10.1038/s41419-026-08433-6)

Full unedited gel for Figure 3G

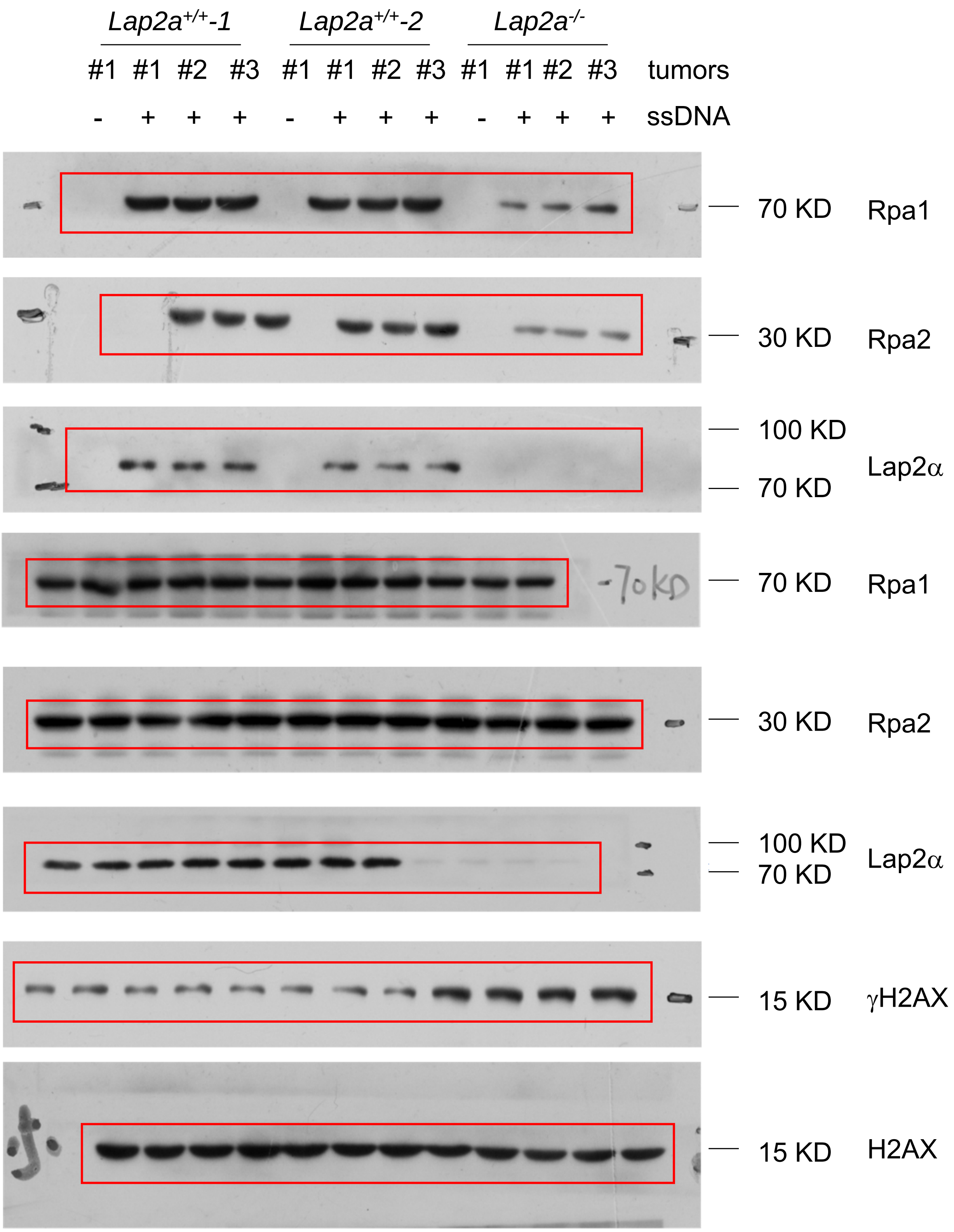

Full unedited gel for Figure 4F

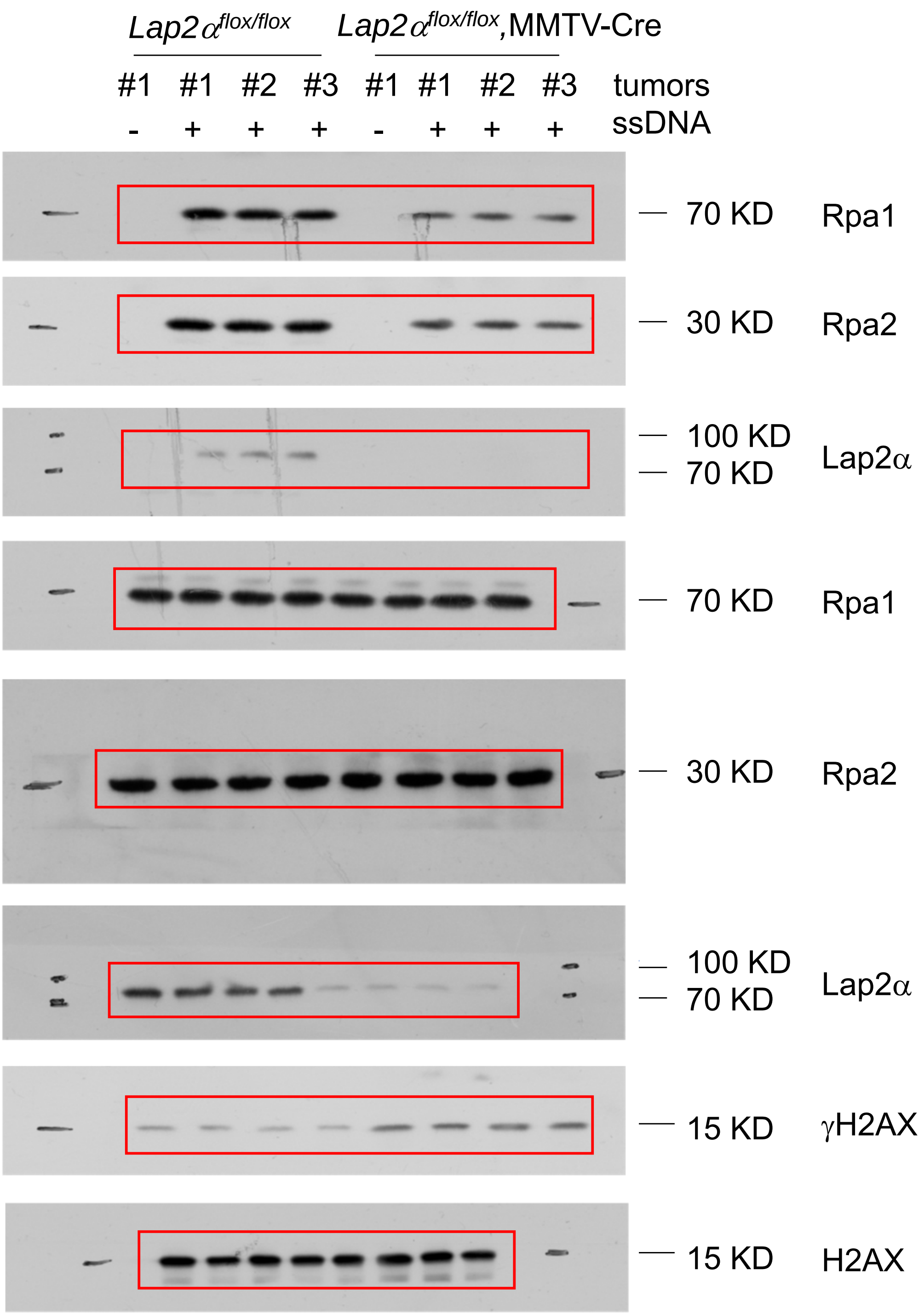

Full unedited gel for Figure 5A

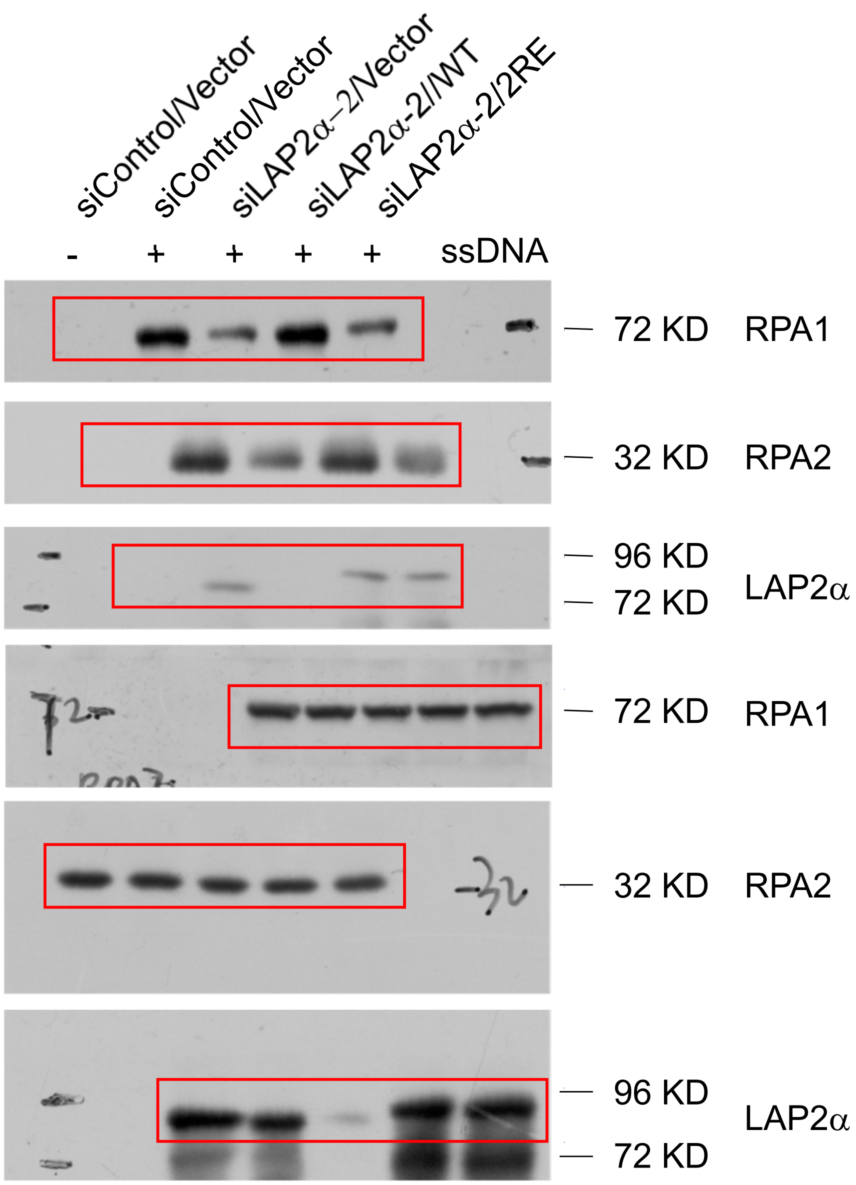

Full unedited gel for Figure 6D

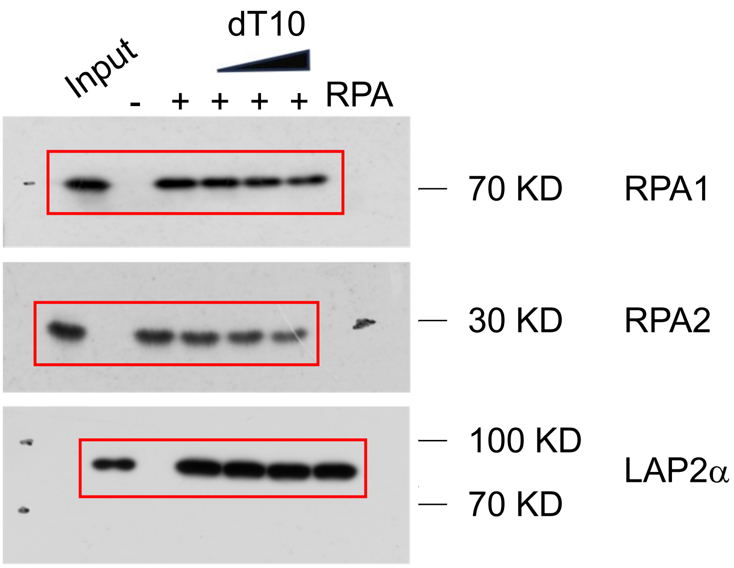

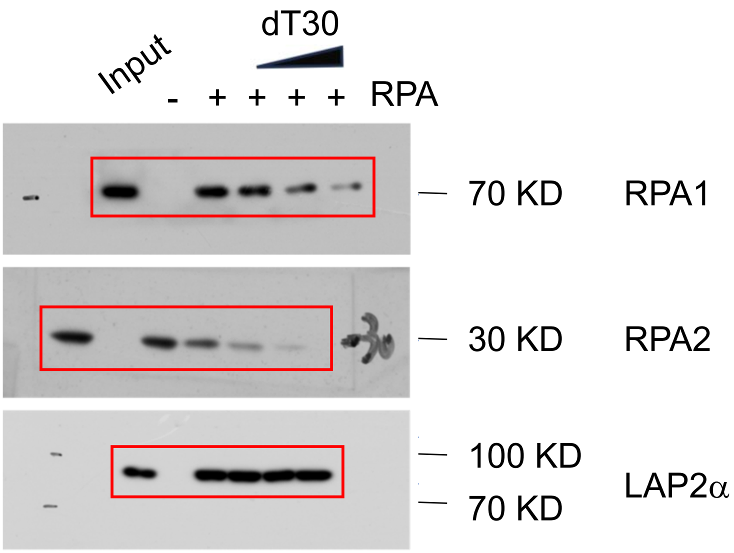

Full unedited gel for Figure S3E

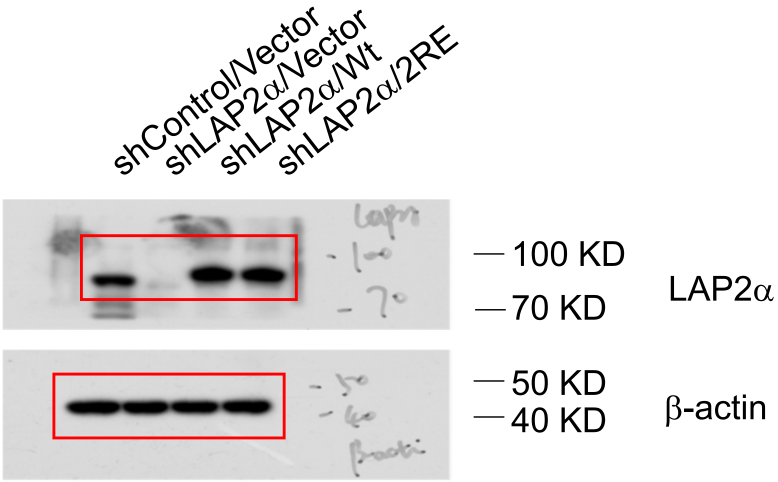

Full unedited gel for Figure S4F

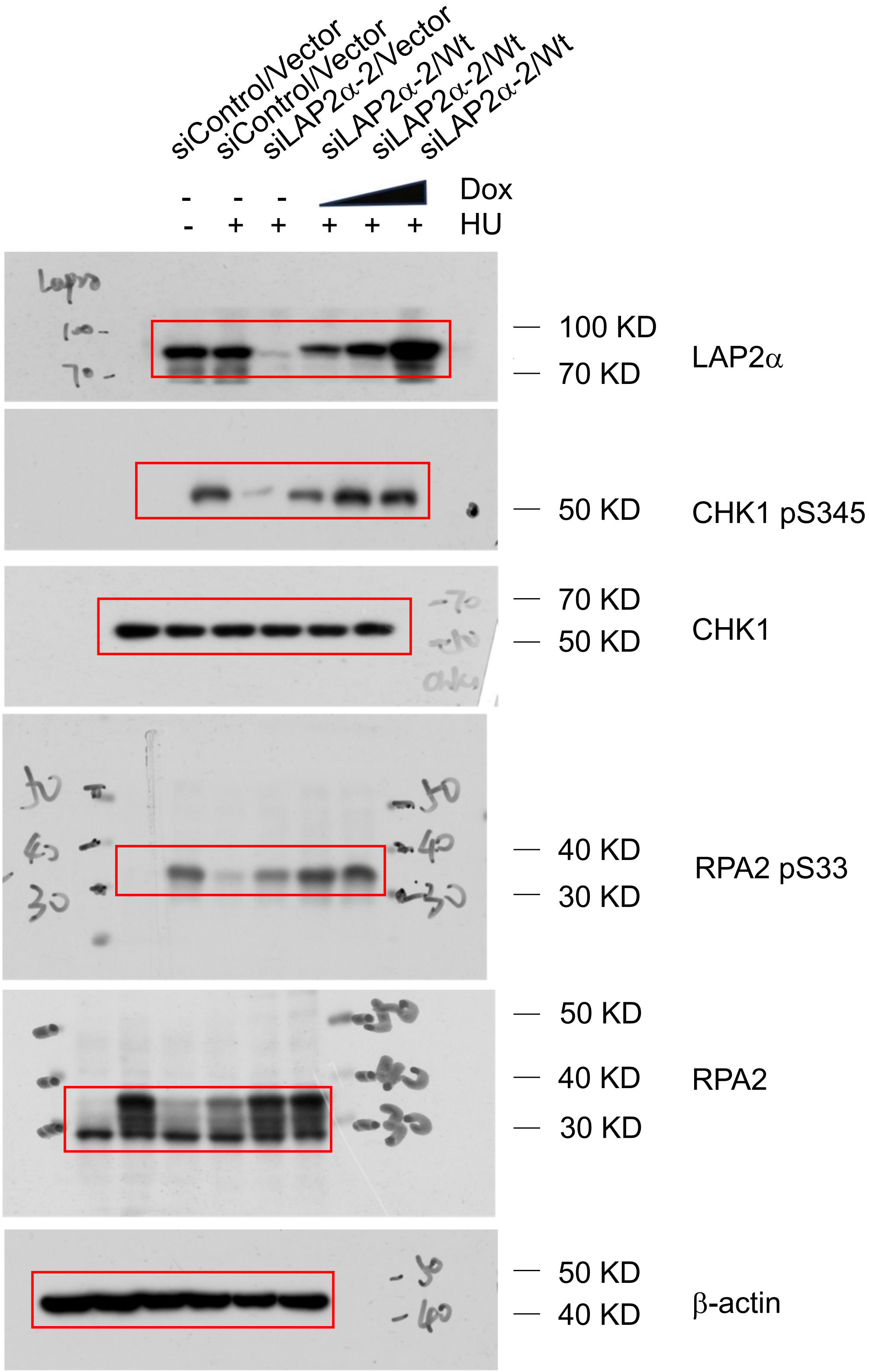

Supplement: Supplementary file 3 — Full and uncropped western blots [file 41419_2026_8433_MOESM3_ESM.pdf]
